# Selection for somatic escape variants in *SERPINA1* in the liver of patients with alpha-1 antitrypsin deficiency

**DOI:** 10.1038/s41588-025-02125-1

**Published:** 2025-03-10

**Authors:** Natalia Brzozowska, Lily Y. D. Wu, Vera Khodzhaeva, William J. Griffiths, Adam Duckworth, Hyunchul Jung, Tim H. H. Coorens, Yvette Hooks, Joseph E. Chambers, Peter J. Campbell, Stefan J. Marciniak, Matthew Hoare

**Affiliations:** 1 Wellcome Trust Sanger Institute, Hinxton, UK; 2 Cambridge Institute for Medical Research, Keith Peters Building, Cambridge Biomedical Campus, University of Cambridge, Cambridge, UK; 3 Department of Medicine, University of Cambridge, Cambridge, UK; 4 Department of Pathology, Addenbrooke’s Hospital, Cambridge, UK; 5 Broad Institute of MIT and Harvard, Cambridge, MA USA; 6 Early Cancer Institute, University of Cambridge, Cambridge, UK

**Keywords:** DNA sequencing, Liver diseases

## Abstract

Somatic variants accumulate in non-malignant tissues with age. Functional variants, leading to clonal advantage of hepatocytes, accumulate in the liver of patients with acquired chronic liver disease (CLD). Whether somatic variants are common to CLD from differing etiologies is unknown. We analyzed liver somatic variants in patients with genetic CLD from alpha-1 antitrypsin (A1AT) deficiency or hemochromatosis. We show that somatic variants in *SERPINA1*, the gene encoding A1AT, are strongly selected for in A1AT deficiency, with evidence of convergent evolution. Acquired *SERPINA1* variants are clustered at the carboxyl terminus of A1AT, leading to truncation. In vitro and in vivo, C-terminal truncation variants reduce disease-associated Z-A1AT polymer accumulation and disruption of the endoplasmic reticulum, supporting the C-terminal domain swap mechanism. Therefore, somatic escape variants from a deleterious germline variant are selected for in A1AT deficiency, suggesting that functional somatic variants are disease-specific in CLD and point to disease-associated mechanisms.

## Abstract (web summary)

The expansion of clones with distinct *SERPINA1* somatic mutants in the livers of alpha-1 antitrypsin deficiency (A1AT) patients is consistent with convergent evolution. These variants interfere with the auto-polymerization and intra-ER accumulation of the Z-A1AT protein, thus highlighting potentially targetable domains.

## Main

CLD leading to cirrhosis accounts for 1 in 25 deaths globally^1^. The etiology of CLD is changing, with fewer cases attributable to viral hepatitis but an increased incidence of obesity and type 2 diabetes driving a worldwide increasing prevalence of metabolic dysfunction-associated steatotic liver disease (MASLD). In addition to these diseases, significant numbers of CLD cases relate to germline genetic disorders, including hemochromatosis and A1AT deficiency^2^. Hemochromatosis, common in patients in northern Europe, most frequently results from inherited mutations within *HFE* or other genes involved in iron absorption and metabolism^3^, leading to the deposition of hepatocellular iron and subsequent cellular stress. A1AT deficiency results from inherited homozygous E366K (E342K in the mature protein) mutations in the *SERPINA1* gene, commonly known as the Z-variant of the A1AT protein^4^. Unlike the wild-type M-A1AT protein, which is secreted into the serum to inhibit neutrophil elastase, the Z-variant polymerizes in the hepatocyte endoplasmic reticulum (ER), leading to ER stress and cell death. There are currently no treatment options for patients with progressive PiZZ A1AT deficiency, aside from liver transplantation.

Somatic acquisition of functionally advantageous variants with subsequent clonal expansion points to the differential selection pressure that operates within the diseased microenvironment. In our previous work on MASLD and alcohol-related liver disease, we identified convergent evolution of functional variants in metabolism genes, including *FOXO1, CIDEB* and *GPAM*. These drive clonal hepatocyte expansion, probably through modulation of carbohydrate and lipid metabolism^5^, which are known to be dysregulated in MASLD. Loss of function in the homologous genes leads to clonal expansion of hepatocytes in mouse models of MASLD^6^; common germline variants of *GPAM*^7,8^ or rare predicted loss-of-function germline variants of *CIDEB*^9^ protect against the development of MASLD. However, it is likely that different diseases leading to specific microenvironmental stressors will drive differential selective advantage of driver variants in similar contexts: liver tissues from patients in the southern United States, predominantly with viral hepatitis, develop a different spectrum of somatic variants^10^. Therefore, the identification of advantageous somatic variants that arise in specific disease contexts potentially points to underlying functional mechanisms. For variants protective at both cellular and systemic levels, mimicry would be a rational therapeutic strategy, especially in liver diseases for which no therapeutics currently exist.

In this study, we explored somatic variants and clonal dynamics in the liver of patients with end-stage CLD caused by hemochromatosis and A1AT deficiency. In a group of patients with background demographics similar to our previous cohort with MASLD^5,11^, but with CLD of distinct etiology, we reasoned that disease-specific pressure would drive the expansion of clones carrying distinct functional variants in genetic CLD: both diseases have pathological mechanisms that do not overlap with each other or with steatotic liver disease (SLD).

## Results

### Somatic variants in the liver in genetic CLD

**Fig. 1:**
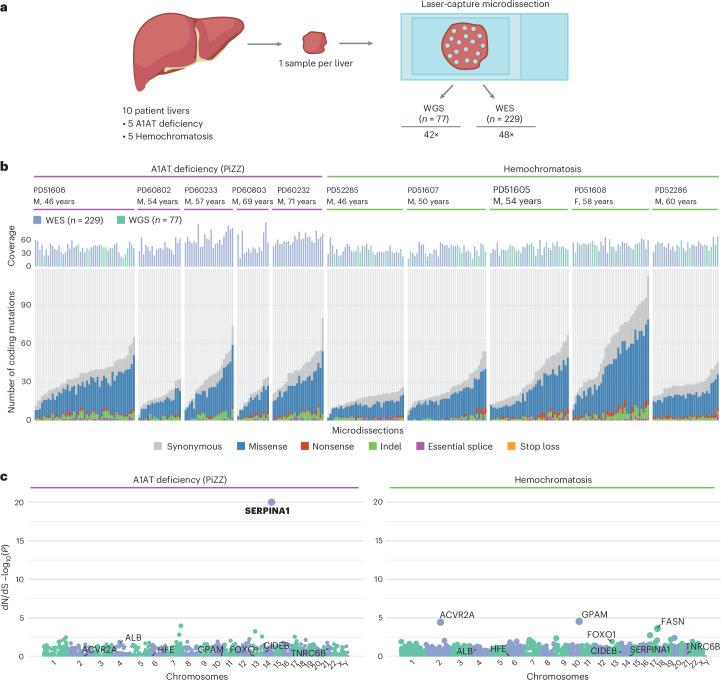
Liver somatic variants in A1AT deficiency and hemochromatosis. **a**, Overview of the hierarchical experimental design: ten livers from patients with A1AT deficiency and hemochromatosis were sampled. These samples underwent LCM to generate 306 individual microdissections; isolated DNA was then sequenced using WGS or WES approaches. **b**, Top panel, mean sequencing coverage in each microdissection. Donors (M, male; F, female) are ordered by disease and age. The number of SNVs and indels detected per microdissection within the coding exome across WGS (*n* = 77) and WES (*n* = 229) data (for five samples for which dual WGS and WES were performed, only WGS calls are shown). Variants are colored by type. **c**, Genes under positive selection in A1AT deficiency and hemochromatosis cohorts. Manhattan plots showing the distribution of *P* values testing for gene-level non-synonymous variants in A1AT deficiency (left panel) and hemochromatosis (right panel). Genes found to be under positive selection in A1AT deficiency, hemochromatosis or, previously, in SLD are labeled. Genes are ordered by genomic position, and those with significant *q* values (<0.1) are highlighted in bold. Source data

**Extended Data Fig. 1:**
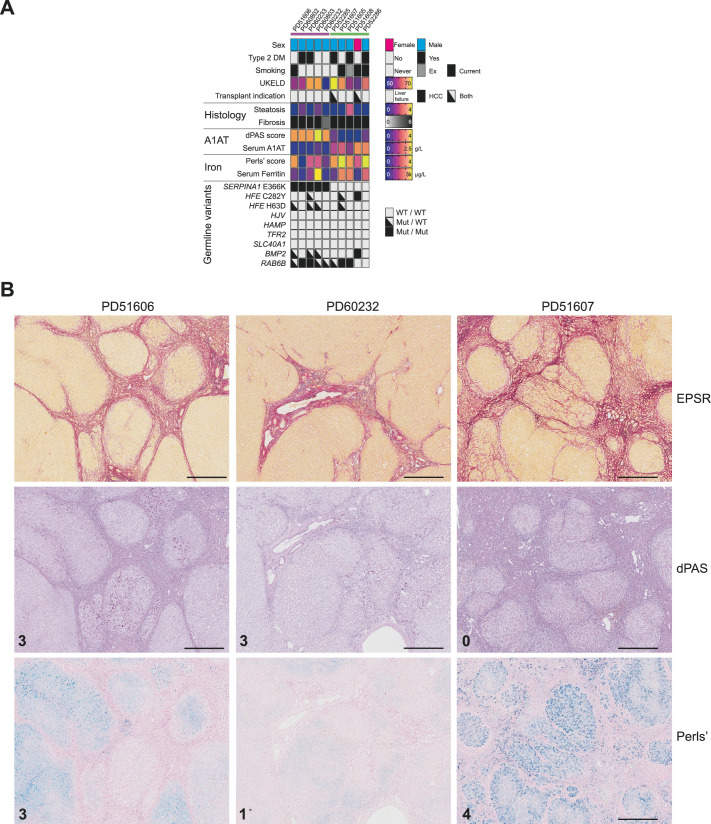
Clinical features and histopathology of patients included in the study. (**a**) Genomic and clinicopathological features of patients with alpha-1 anti-trypsin (A1AT) deficiency (purple header bar, n = 5) and haemochromatosis (green header bar, n = 5) included in the study. Germline variants were called from the exome or whole genome sequencing and reported for genes previously associated with either A1AT-deficiency or haemochromatosis. (**b**) Example photomicrographs of serial liver sections stained for enhanced picosirius red (EPSR, upper panels), diastase-Periodic Acid Schiff (dPAS, middle panels) or Perls’ stain (lower panels) from three patients included in the study. Numbers on the panels are the dPAS granules / globules score (0–4) or hepatocellular iron deposition score (0–4) from global assessment of liver histology. Scale bars 1 mm.

**Extended Data Fig. 2:**
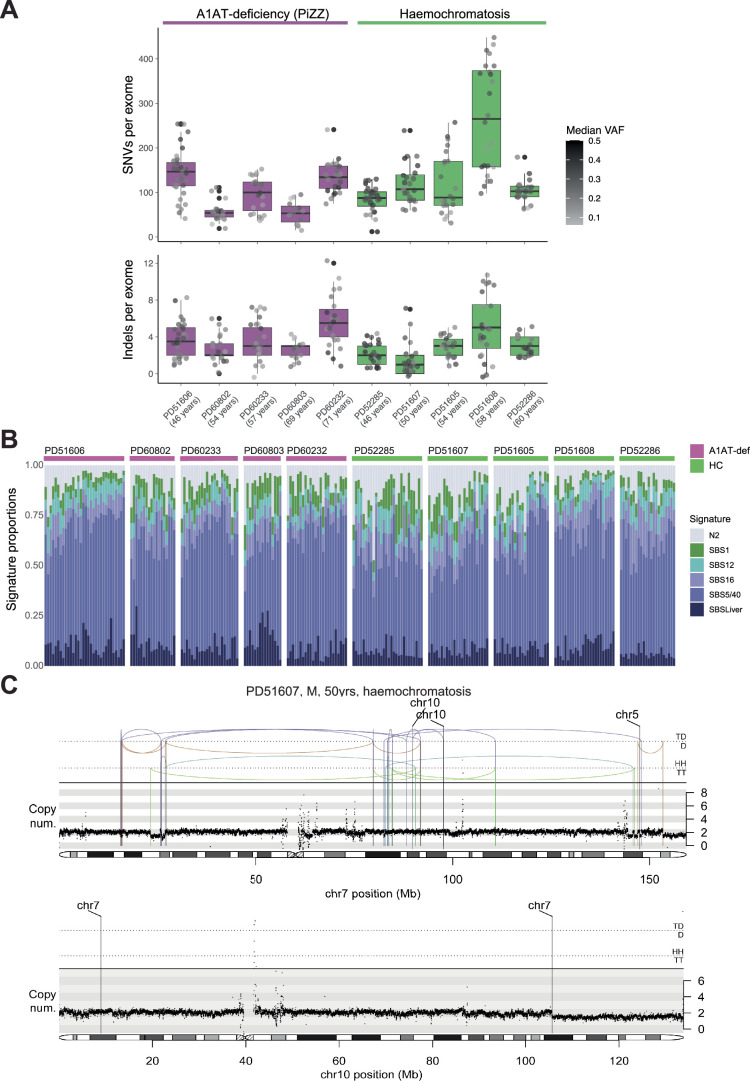
Mutational burden and large-scale rearrangements identified in the liver from A1AT-deficiency and haemochromatosis. (**a**) Upper panel: burden of single-nucleotide variants (SNVs) per exome, corrected by sensitivity of mutation detection. Each box plot represents a patient (n = 10 patients; 305 microdissections) and each dot represents one laser-capture microdissected sample. The grey-to-black intensity of the points reflects the median variant allele fraction (VAF) of mutations in each microdissection. Boxes in the box plots indicate median and interquartile range; whiskers denote range. Lower panel: burden of indel variants per exome (n = 10 patients; 305 microdissections). (**b**) Relative proportional contributions of the six most abundant SBS signatures across A1AT-deficiency (purple-headed columns) and haemochromatosis (green-headed columns) donors. Each bar represents a laser capture microdissection, in ascending order of the number of coding mutations. (**c**) Chromothripsis involving chromosomes 5 and 10 (upper panel) and chromosomes 7 and 10 (lower panel), observed in patient with haemochromatosis (PD51607). Black points represent corrected read depth along the chromosome. Lines and arcs represent structural variants, coloured by the orientation of the joined ends (purple, tail-to-tail inverted; brown, head-to-head inverted; turquoise, tandem-duplication-type orientation; green, deletion-type orientation).

We obtained explanted liver tissue from five patients with homozygous PiZZ A1AT deficiency (Supplementary Table 1 and Extended Data Fig. 1) and five patients with hemochromatosis; all had significant hepatocellular iron deposition and included one patient with homozygous C282Y mutations, one with compound C282Y/H63D mutations and three with hemochromatosis of uncertain cause (Supplementary Table 2) without risk factors for secondary hemochromatosis. All were undergoing liver transplantation for liver failure, hepatocellular carcinoma or both. To identify somatic variants, we initially performed whole-genome sequencing (WGS) in one patient with A1AT deficiency and four with hemochromatosis but thereafter used only whole-exome sequencing (WES) in the remaining cases to focus on coding variation. We performed laser-capture microdissection (LCM) and WGS or WES (Fig. 1a) of 306 laser microdissections of liver tissue, achieving a mean coverage of 42× for WGS and 48× for exome sequencing (Fig. 1b). To consider exome and genome datasets together, we reported the number of exonic coding mutations (Fig. 1b). There was considerable heterogeneity in variant burden both between and within patients (Extended Data Fig. 2a) but no significant differences between A1AT deficiency, hemochromatosis or previously sequenced liver samples from patients with SLD^5^ and no differential representation of mutational signatures (Extended Data Fig. 2b). Copy number changes and structural variants (SVs) (called in samples undergoing WGS) occurred in moderate numbers across patients with hemochromatosis and the one patient with A1AT deficiency, with WGS data (Extended Data Fig. 2a). One clone demonstrating chromothripsis involving chromosomes 7 and 10 was detected in the liver of a patient with hemochromatosis undergoing transplantation for liver failure without hepatocellular carcinoma (Extended Data Fig. 2c), accounting for 30 out of 48 of SVs and 30 out of 34 copy number variants in that donor.

We analyzed which genes were under positive selection by applying an implementation of the ratio of non-synonymous to synonymous mutation rates (dN/dS) while accounting for sequence composition, trinucleotide mutational biases and gene-specific variation in mutation rates^12^. Across all protein-coding genes, we found that somatic variants in *SERPINA1* were under strong positive selection (dN/dS, ~783 for truncating, ~1,772 for insertions and deletions (indels); *q* < 2 × 10^−16^) (Fig. 1c) in patients with A1AT deficiency but not in patients with hemochromatosis (dN/dS, 0 for truncating variants; 95% CI, 0.0–405.6; *q* = 1) or SLD^5,11^ (dN/dS, 0; 95% CI, 0.0–71.0; *q* = 1). We did not identify any enrichment for somatic variants in any gene previously associated with iron metabolism^3^, including *HFE*, in either cohort. Furthermore, no acquired variants were significantly positively selected for in the patients with hemochromatosis; however, this may reflect a lack of statistical power given the small number of patients examined. Genes previously found to be under positive selection in liver tissue from patients with SLD^5^ or liver cancer^13^ did not appear to be under positive selection in liver tissue from either the A1AT deficiency (*CIDEB* dN/dS for missense, 30.5 (95% CI, 1.7–142.8; *q* = 1); *FOXO1* dN/dS, 0.0 (95% CI, 0.0–18.2; *q* = 1); *GPAM* dN/dS, 0.0 (95% CI, 0.0–16.9; *q* = 1)) or hemochromatosis cohorts (*CIDEB* dN/dS, 24.9 (95% CI, 1.4–110.4; *q* = 1); *FOXO1* dN/dS, 22.4 (95% CI, 5.5–58.7; *q* = 1); *GPAM* dN/dS, 13.8 (95% CI, 2.3–42.9; *q* = 0.35)). Therefore, liver diseases of differing etiology drive the expansion of hepatocyte clones with distinct somatic variants.

### Convergent evolution of *SERPINA1* variants in A1AT deficiency

**Fig. 2:**
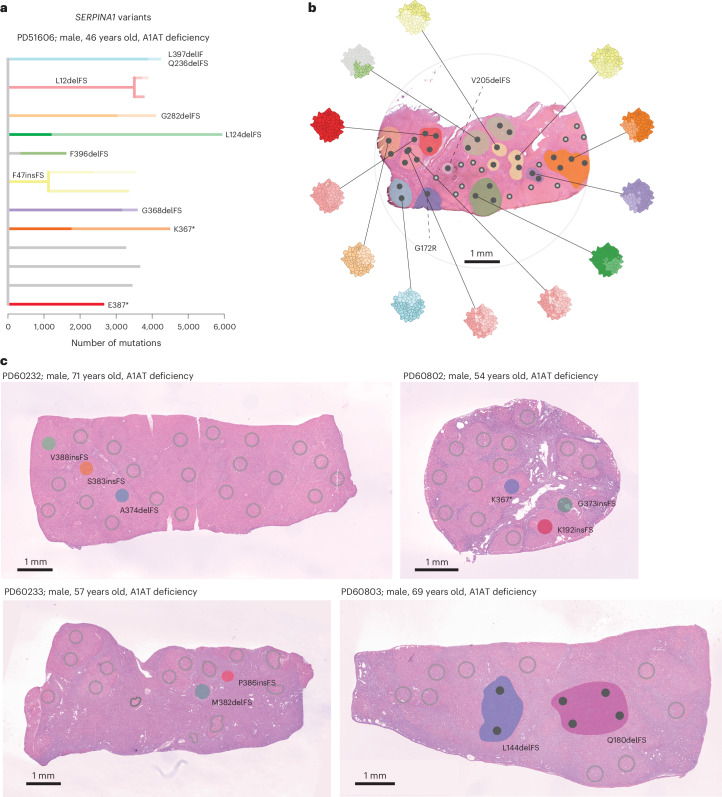
Convergent evolution of somatic variants of *SERPINA1* in A1AT deficiency. **a**, Phylogenetic tree of clonal structure from liver sample PD51606, with colored branches showing independently acquired *SERPINA1* mutations. **b**, Spatial mapping of the clones from the phylogenetic tree onto an H&E-stained photomicrograph of the patient’s liver biopsy, with *SERPINA1* mutant clones colored to match the tree shown in **a**. Each dot signifies a microdissection, with solid black dots indicating LCM microdissections harboring a *SERPINA1* mutation. For WGS samples, clonal cell fraction is depicted, with a fully filled disk signifying all clones detected in the LCM sample and *SERPINA1* mutant cell fraction depicted by the degree of disk filling. Dashed lines indicate *SERPINA1* mutations identified exclusively in WES samples. Nodules are color-highlighted, whereby all microdissections within the nodule contain the same *SERPINA1* mutant clone. **c**, Spatial location of clones with *SERPINA1* variants in the H&E-stained liver of the remaining four patients with A1AT deficiency (colored area), in addition to *SERPINA1* wild-type clones (gray circles).

**Extended Data Fig. 3:**
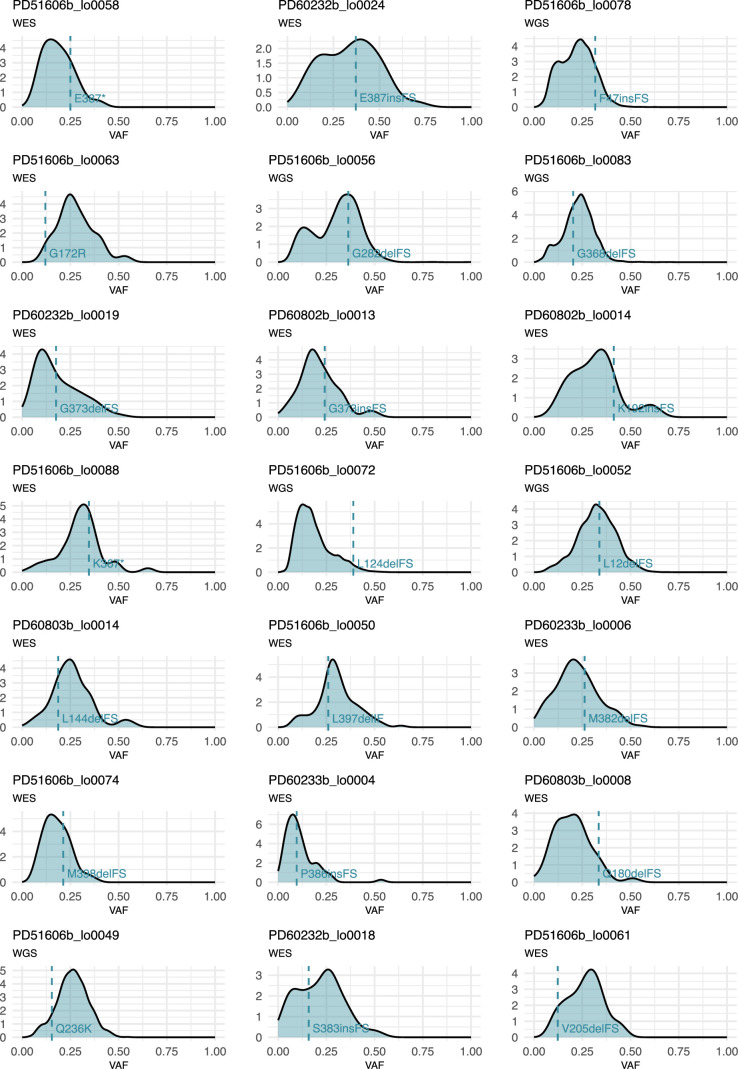
Variant allele fraction (VAF) density plots of LCM microdissections depicting VAFs of *SERPINA1* mutations. Dashed line drawn at the mean VAF of SERPINA1 mutation in that sample. WES, whole exome sequencing. WGS, whole genome sequencing.

In the five patients with A1AT deficiency, we observed 17 indels, two missense and three nonsense mutations in *SERPINA1*. All five patients had genetic evidence of convergent evolution, with multiple independent clones containing distinct *SERPINA1* variants within the same piece of randomly selected liver tissue (Fig. 2). One patient had 11 independent clones with *SERPINA1* variants within 0.3 cm^2^ of liver tissue, with one clone containing frameshift deletions in both alleles of the gene (Fig. 2a,b). The other four patients had two or three clones with independent *SERPINA1* variants (Fig. 2c); most non-synonymous mutations in *SERPINA1* in the A1AT deficiency cohort had variant allele fractions (VAFs) between 0.2 and 0.4, well within the VAF distribution for passenger mutations in the respective samples, indicative of being heterozygous rather than homozygous, in the major clone (Extended Data Fig. 3). Therefore, heterozygous somatic variants in *SERPINA1* provide selective advantage in A1AT deficiency, which only develops in patients with homozygous germline Z variants of *SERPINA1*.

**Fig. 3:**
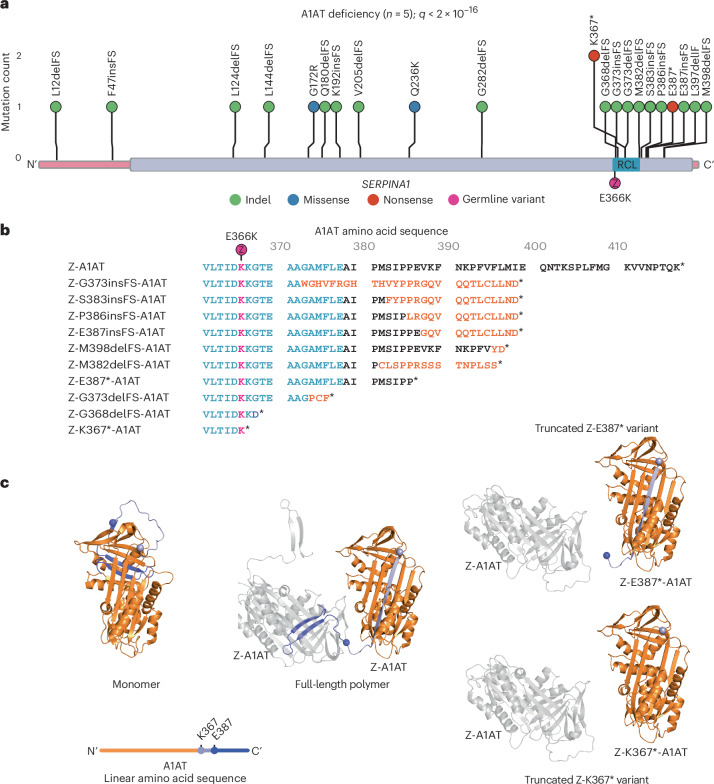
Recurrent variants of *SERPINA1* are predicted to lead to C-terminal protein truncations. **a**, Distribution of non-synonymous variants detected in *SERPINA1* in liver samples from patients with A1AT deficiency (*n* = 5) with gene-level *q* value corrected for multiple hypothesis testing reported. The coding sequence of the gene is represented on the *x* axis, with exons in pink and protein domains in purple. Position of the germline PiZ variant (E366K) shown in magenta; RCL, reactive center loop. **b**, Effect of C-terminal variants on Z-A1AT protein sequence; sequence alignment of Z-A1AT amino acids 361–418. The reactive center loop is highlighted in blue; amino acid sequence modified by frameshift in orange; E366K (PiZ) variant indicated in magenta; stop codon denoted by *. **c**, Left, cartoon representing the crystal structure of native A1AT derived from Protein Data Bank (PDB 1QLP)^15^. The position of residues K367 (light purple) and E387 (blue) is shown by colored spheres, with downstream residues colored similarly. A linear schematic of the protein shows relative mutation positioning using the same color code (upper). Middle, the crystal structure of two protomers in a Z-A1AT polymer from PDB 3T1P^16^, showing the insertion of donor protomer residues downstream of K367 (light purple sphere) into the acceptor protomer. Right, structural model of two protomers in a polymer (PDB 3T1P) highlighting the incompatibility of Z-K367* and Z-E387* C-terminal donation to a polymer.

**Extended Data Fig. 4:**
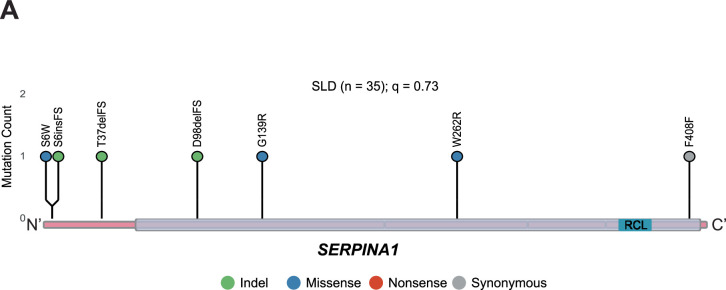
Somatic variants in *SERPINA1* in the liver of patients with MASLD. (**a**) Distribution of variants detected in *SERPINA1* in liver samples from patients with SLD (n = 35) from Ng et al. Coding sequence of the gene represented on the x-axis, with exons in pink and protein domains in purple. Serpin, serine protease inhibitor domain; RCL, reactive centre loop.

We were interested in exploring the potential protein-coding effects of the somatic variants in *SERPINA1*. Notably, the identified variants in the A1AT deficiency cohort showed strong clustering of truncating variants within the last exon of *SERPINA1* (Fig. 3a), not seen in the previous SLD cohort (Extended Data Fig. 4): among 29 patients with SLD, three patients had a heterozygous PiMZ *SERPINA1* genotype with no coding variants in *SERPINA1* in these liver samples. In the A1AT-deficiency cohort, all frameshift indels and nonsense mutations in the last exon are predicted to result in the loss of at least 19 amino acids from the C-terminus of the encoded protein in addition to partial modification of amino acid sequence in the case of frameshift indels (Fig. 3b). The recurrence and convergent evolution of similar variants suggests that acquired C-terminal truncating *SERPINA1* variants provide a selective advantage to hepatocytes in A1AT deficiency through common functional mechanisms.

### C-terminal clustering of somatic *SERPINA1* variants

**Extended Data Fig. 5:**
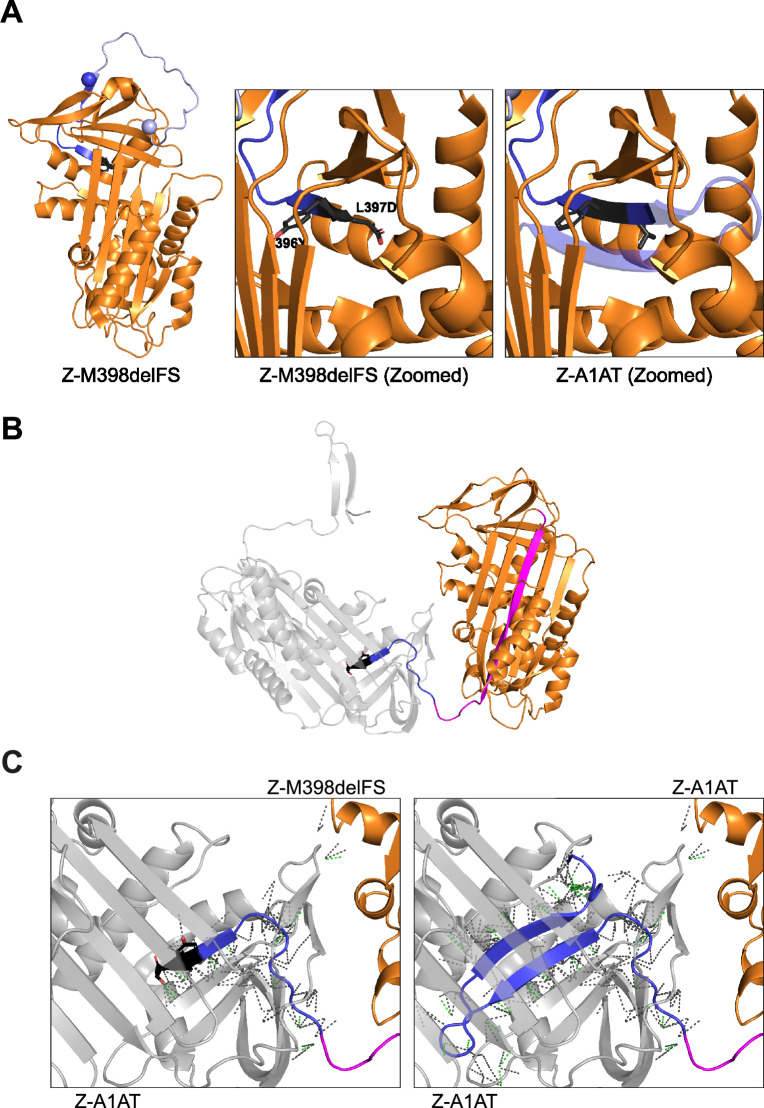
Structural prediction of the influence of Z-M398delFS somatic mutation. (**a**) Left: Cartoon of the primary sequence of the somatic A1AT mutant Z-M398delFS mapped as a portion of the structure of native full length A1AT derived from Protein Data Bank (PDB):1QLP17. Magenta and blue regions reflect portions of the protein sequence lost in the somatic mutants K367* and E387* respectively (mutation sites modeled as spheres), as shown in Fig. 3c. Centre: Zoomed section of A1AT beta-sheet C showing the missense F396Y and L397D residues of Z-M398delFS in black with amino acid side chains rendered. Right: A comparative zoom of full length A1AT showing the side chains of native F396 and L397, with structure downstream of the Z-M398delFS premature STOP codon rendered opaque. (**b**) Cartoon of the primary sequence of A1AT Z-M398delFS mapped onto the crystal structure of a Z-A1AT polymer from PDB:3T1P18, where Z-M398delFS is displayed as the donor protomer and the acceptor Z-protomer is grey. Residues downstream of K367 and E387 are shown in magenta and blue respectively. F396Y and L397D are shown in black. (**c**) Left panel: Zoomed polymer interface shown from (**b**) showing contacts between protomers as dashed lines (polar contacts are green, others are grey). Right panel: A corresponding map of inter-protomer contacts between two molecules of full-length Z-A1AT.

Somatic variants leading to truncation (Fig. 3c, right) or sequence changes (Extended Data Fig. 5) in the C-terminus of A1AT have the potential to disrupt both native folding and polymerization of A1AT. The recurrent *SERPINA1* variants affect the reactive center loop and C-terminus of A1AT, previously shown to mediate homopolymerization of Z-A1AT (Fig. 3c)^14^. In its native fold, the C-terminal 26 amino acids of A1AT form a β-hairpin that inserts into and completes β-sheet B, with β-sheet A adopting a five-stranded conformation^15^ (Fig. 3c, left). Polymers arise through the completion of β-sheet B in an ‘acceptor’ protomer by the C-terminal β-hairpin of a second ‘donor’ protomer^14,16^. This causes the acceptor molecule to adopt a hyper-stable, six-stranded β-sheet, which is a conformation that translocates its own orphaned C-terminus to the opposite pole of the molecule where it can act as a donor to drive polymer extension (Fig. 3c, middle). Therefore, somatic variants that impose truncation or sequence changes in the C-terminus have the potential to disrupt polymerization of Z-A1AT through lost or reduced ability (Fig. 3c and Extended Data Fig. 5) to donate the C-terminus into neighboring Z protomers, but should not affect their ability to act as ‘acceptors’.

### C-terminal truncation variants of Z-A1AT rescue ER disruption

**Fig. 4:**
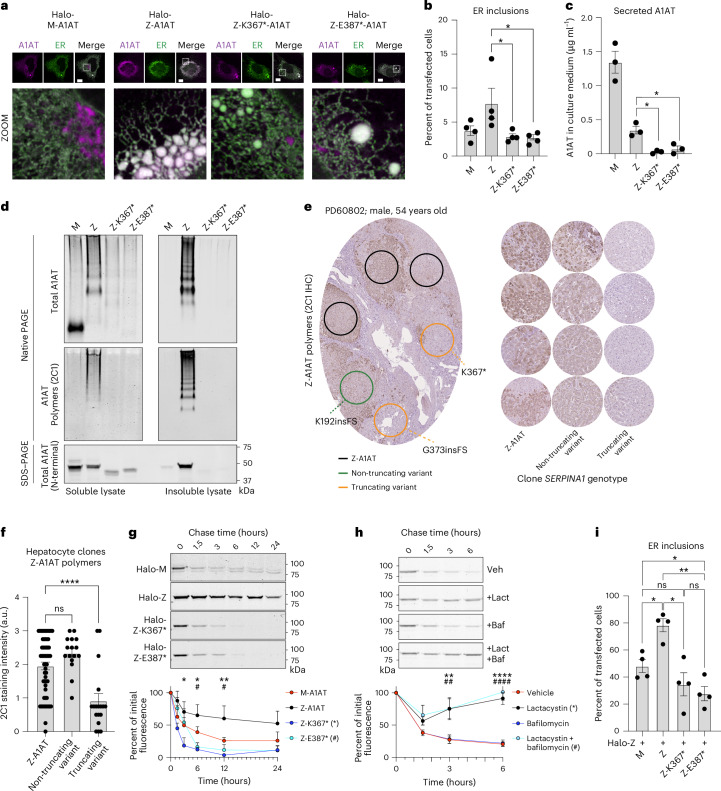
C-terminal truncation variants of *SERPINA1* inhibit Z-A1AT polymer accumulation and ER disruption. **a**, Fluorescence micrographs of CHO cells expressing indicated HaloTagged variants of A1AT (magenta) with the ER marker protein (moxGFP-KDEL (green)). Dashed line boxes indicate the area expanded in the zoomed-in images. Scale bar, 10 μm. **b**, ER inclusions (characterized by a region of fragmented tubular ER network) in CHO cells as in **a**; Z versus Z-K367*, *P* = 0.0283; Z versus Z-K387*, *P* = 0.0248. **c**, Levels of secreted A1AT from CHO cells expressing indicated A1AT variants; Z versus Z-K367*, *P* = 0.0053; Z versus Z-K387*, *P* = 0.017. **d**, SDS–PAGE and native PAGE immunoblotting of soluble and insoluble cell lysate fractions from an equivalent cell mass of CHO cells expressing indicated A1AT variants. Native PAGE immunoblotting was performed using antibodies recognizing total (a0409) and polymerized (mAB_2C1_) A1AT. SDS–PAGE immunoblotting was performed using an N-terminal directed antibody against A1AT present in all variants (MA5-15521). **e**, Left, exemplar photomicrograph of liver tissue immunohistochemistry for Z-A1AT polymers. Circles indicate regions with microdissected hepatocyte clones analyzed by WES or WGS (as in Fig. 1) in an adjacent section with the indicated *SERPINA1* genotype; right, exemplar Z-A1AT polymer immunohistochemistry from regions with the indicated *SERPINA1* genotype across the A1AT-deficiency cohort. **f**, Quantification of Z-A1AT polymer staining intensity (as in **e**) of 91 microdissected areas from five patients with A1AT deficiency; Z-A1AT versus non-truncating variant, *P* = 0.202; Z-A1AT versus truncating variant, *P* < 0.0001; ns, not significant. **g**, Pulse–chase time course of CHO cells expressing indicated HaloTagged A1AT variants using the HaloTag ligand. Quantitation of HaloTag-band intensity is plotted. * and # denote the significance of Z versus Z-K367* (*P* = 0.012, 0.010 and 0.005 at 3, 6 and 12 h, respectively) or Z versus Z-E387* (*P* = 0.805, 0.020 and 0.022 at 3, 6 and 12 h, respectively), respectively. **h**, Pulse–chase of HaloTagged Z-K367* in the presence of dimethylsulfoxide (vehicle), lactacystin (proteosome inhibitor, 5 µM), bafilomycin (lysosomal inhibitor, 100 nM) or both. * and # denote significance of vehicle versus lactacystin (*P* = 0.597, 0.002 and <0.0001 at 1.5, 3 and 6 h, respectively) or vehicle versus both inhibitors (*P* = 0.172, 0.002 and <0.0001 at 1.5, 3 and 6 h, respectively), respectively. **i**, Proportion of CHO cells displaying ER inclusions when co-expressing HaloTagged Z-A1AT with untagged variants of A1AT (Z + M versus Z + Z, *P* = 0.027; Z + Z versus Z + Z-K367*, *P* = 0.012; Z + Z versus Z + Z-K387*, *P* = 0.002; Z + M versus Z + Z-K387*, *P* = 0.033); *n* = 4 (**a**, **b** and **i**) or *n* = 3 (**c**, **d**, **g** and **h**) biological replicates for all conditions. Columns (**b**, **c**, **f** and **i**) and points (**g** and **h**) represent means ± s.e.m. Analyses by one-way (**b**, **c**, **f** and **i**) or two-way (**g** and **h**) ANOVA with Šidák’s correction for multiple hypothesis testing; ns, not significant; * *P* ≤ 0.05; ** *P* ≤ 0.01; **** *P* ≤ 0.0001.

To explore potential mechanisms, including polymerization, underlying the selective advantage of Z-A1AT C-terminal truncation variants to hepatocytes, we modeled these in vitro. We studied two variants with acquired premature stop codons: Z-K367* and Z-E387* (Fig. 3b,c). To study the cellular localization of these Z-A1AT variants, we conducted live-cell imaging of HaloTagged A1AT in an established Chinese hamster ovary (CHO) cell model that lacks human A1AT expression^17,18^. Both M-A1AT and Z-A1AT colocalized with the ER marker protein moxGFP-KDEL, while only the M form also localized to perinuclear structures resembling the Golgi apparatus. Expression of Z-A1AT promotes fragmentation of the ER network, giving rise to punctate ER inclusions (Fig. 4a). We quantified ER inclusion formation in cells expressing HaloTagged M, Z, Z-K367* or Z-E387* variants. Z-A1AT showed a greater propensity to form ER inclusions than M-A1AT (Fig. 4a,b), as reported previously^18,19^; by contrast, Z-K367* and Z-E387* led to a similar propensity to form ER inclusions as seen with wild-type M-A1AT expression (Fig. 4a,b). Therefore, the C-terminal truncation variants of Z-A1AT show reduced accumulation within, and disruption of, the ER.

**Extended Data Fig. 6:**
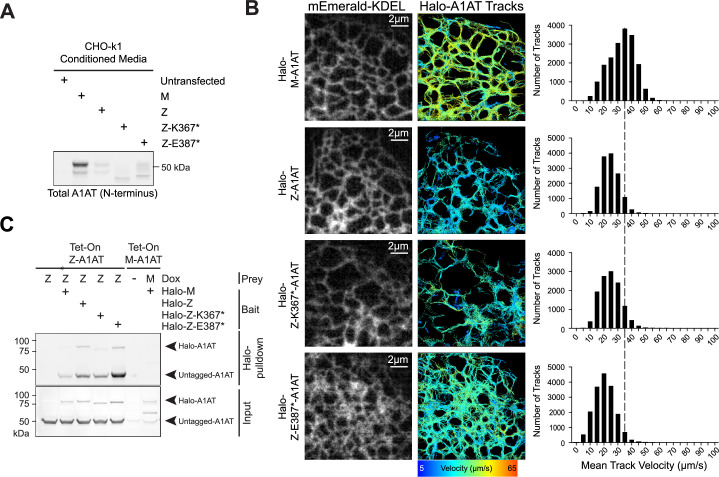
Somatic C-terminal truncation variants of *SERPINA1* do not rescue A1AT-secretion but have a short protein half-life. (**a**) Western blot analysis of A1AT secretion by immunoblotting of media conditioned for 24 h by CHO cells transfected with the indicated A1AT-variant constructs. Immunoblotting was performed using a monoclonal antibody raised against an N-terminal fragment of human α1-antitrypsin (MA5-15521). Images representative of n = 3 biologically independent experiments. (**b**) Micrographs of the ER marker protein mEmerald-KDEL expressed in COS7 cells (left images) with accompanying single-particle tracks of HaloTagged variants of A1AT labelled with PAJF646, colour coded by mean velocity (right images). Frequency distribution histograms show mean velocity of tracks analysed. A dashed line denotes the median velocity of M-A1AT. For each condition over 10,000 particle tracks were analysed across a minimum of 17 cells from 3 repeats. (**c**) CHO cells conditionally-expressing untagged Tetracycline-inducible (Tet-ON) Z-A1AT were transfected with expression vectors encoding HaloTagged variants of A1AT, 2 h prior to 1 µg/mL doxycycline treatment. Input and Halo-affinity purified samples were then separated by SDS-PAGE prior to immunoblotting with antibody recognising total (MA5-15521) A1AT. Images representative of n = 3 biologically independent experiments.

The localization of the M variant apparently to the Golgi apparatus supports the expected secretory trafficking of wild-type HaloTagged M-A1AT, with ER retention of the Z-variant^20^ (Fig. 4a). Despite reduced ER retention, the HaloTagged Z-K367* and Z-E387* forms of A1AT showed no evidence of secretory trafficking: quantitative sandwich ELISA (Fig. 4c) and immunoblotting using an amino-terminal-directed antibody (Extended Data Fig. 6a) for secreted (untagged) A1AT in conditioned media from transfected CHO cell cultures indicated that cells expressing Z-K367* and Z-E387* secrete very little A1AT protein. Therefore, the C-terminal truncation variants of Z-A1AT do not accumulate in the ER but do not restore secretion of A1AT.

We next investigated ER handling of C-terminal truncation A1AT variants by assessing their mobility within the ER lumen, using single-particle tracking (Extended Data Fig. 6b)^21,22^ in COS7 cells. As shown previously, single Z-A1AT particles displayed a significant reduction in velocity compared to M-A1AT (Extended Data Fig. 6b)^18^. Z-K367* and Z-E387* also showed significantly reduced velocity, suggesting that these C-terminal truncation variants do not restore normal ER handling. Reduced mobility of Z-K367* and Z-E387* could indicate either an increase in protein hydrodynamic volume through polymerization or interactions with ER quality-control factors. Therefore, C-terminal truncation variants of A1AT have a reduced propensity to form ER inclusions, but these variants failed to rescue impaired molecular mobility within the ER or trafficking through the secretory pathway.

### C-terminal truncation variants of Z-A1AT prevent polymerization

Based on the predicted protein sequences, we reasoned that the C-terminal truncation variants could impair aberrant polymerization of Z-A1AT. We assessed A1AT polymer formation in CHO cells expressing untagged A1AT variants by polyacrylamide gel electrophoresis under native non-denaturing (native PAGE) and denaturing (SDS–PAGE) conditions, followed by immunoblotting. As Z-A1AT polymers partition between soluble and insoluble pools within the ER^2,18,23,24^, insoluble proteins were first separated from lysates by centrifugation and resuspended in their initial volume to assess stoichiometry with soluble protein. Native PAGE immunoblotting of the soluble A1AT pool showed that M-A1AT migrated predominantly at a low molecular weight, representing folded monomers (Fig. 4d, upper left panel). Z-A1AT separated as a ladder of higher molecular-weight species in soluble and insoluble pools, confirmed as A1AT polymers by the conformation-specific antibody mAb_2C1_ (Fig. 4d, middle panels). The Z-K367* and Z-E387* truncation variants lack β-strands 4B and 5B, required for C-terminal donation in polymerization, and accordingly produced no higher molecular-weight species (Fig. 4d, middle left panel) that react with polymer-specific mAb_2C1_ (Fig. 4d, middle right panel) despite containing the germline E366K Z-variant. Immunoblotting of lysates separated by SDS–PAGE confirmed lower expression of C-terminal truncation variants compared to Z-A1AT, but they predominantly partition to the soluble fraction of lysates, in contrast to Z-A1AT (Fig. 4d, lower panel). Therefore, acquired C-terminal truncation variants of Z-A1AT do not form polymers characteristic of the germline Z-variant of A1AT.

### Hepatocyte clones with C-terminal truncation variants have reduced A1AT polymers

We next explored whether hepatocyte clones that contained *SERPINA1* variants had evidence of reduced polymer formation in vivo. We used immunohistochemistry with the Z-A1AT polymer-specific 2C1 antibody on serial sections from the five liver samples in the A1AT-deficiency cohort, in which we had already conducted spatial DNA sequencing on the adjacent section (Fig. 4e). We then defined areas of hepatocytes with wild-type *SERPINA1*, non-truncating *SERPINA1* variants or C-terminal truncation variants before assessing the intensity of 2C1 immunostaining in those areas. Hepatocytes with non-truncating *SERPINA1* somatic variants had a similar polymer burden to areas with homozygous germline Z-*SERPINA1*, whereas hepatocytes with C-terminal truncation variants of *SERPINA1* showed a significantly lower polymer burden than wild-type hepatocytes (Fig. 4e,f). Therefore, C-terminal truncation and frameshift variants of Z-A1AT have reduced polymer accumulation in vivo compared to germline Z-A1AT. These findings also raise the interesting question of whether proximal non-truncating variants are truly advantageous or whether these represent passenger mutations.

It remained possible that reduced Z-A1AT polymer accumulation could simply reflect lower expression levels of the C-terminal Z-A1AT variants compared to the germline M-A1AT and Z-A1AT variants (Fig. 4d, lower panel) or impaired polymerization through loss of the C-terminal domain swap. As Z-K367* and Z-E387* variants were heterozygous (VAF ≤ 0.5) with native Z-A1AT expressed from the other allele in hepatocyte clones (Extended Data Fig. 3), we explored whether the reduction in A1AT accumulation was a result of their potential to behave as ‘acceptor’ protomers in forming mixed polymers with Z-A1AT but ‘chain-terminate’ onward polymerization through inability to ‘donate’ to the next Z monomer (Fig. 3c). HaloTagged A1AT variants were constitutively expressed in CHO cells that also conditionally express untagged doxycycline-inducible Z-A1AT before affinity purification of the HaloTagged A1AT species (Extended Data Fig. 6c). SDS–PAGE immunoblotting of the input samples showed expression of the higher molecular-weight HaloTagged A1AT variants in addition to the lower molecular-weight untagged Z-A1AT. Affinity purification of HALO and then immunoblotting showed that the HaloTagged Z-A1AT pulled down lower molecular-weight untagged Z-A1AT; similarly, purification of both Z-K367* and Z-E387* variants retained the ability to pull down untagged Z-A1AT, consistent with their ability to bind to native Z-A1AT derived from the unaffected allele. Therefore, although the C-terminal truncation variants do not form homopolymers, they retain the ability to bind to germline Z-A1AT, acting as ‘acceptor’ protomers.

### C-terminal truncation variants of Z-A1AT have a reduced half-life

We explored why C-terminal variants had apparently lower protein expression using a pulse–chase strategy, labeling the HaloTagged A1AT variants in CHO cells with Janelia Fluor reporters that covalently bind to Halo. Consistent with previous data, we found that Z-A1AT had a longer half-life than M-A1AT (Fig. 4g). However, we found that both Z-K367* and Z-E387* variants had a significantly shorter half-life than Z-A1AT. To probe how these C-terminal variants of A1AT undergo degradation, we used small molecule inhibitors of the two major protein degradation pathways: lactacystin as an inhibitor of the proteosome and bafilomycin A1 as an inhibitor of lysosomal function. Lysosomal inhibition had no effect on the half-life of Z-K367*, whereas proteasomal inhibition completely prevented the degradation of Z-K367* (Fig. 4h). Therefore, lower expression of the C-terminal truncation Z-A1AT variants, compared to germline Z-A1AT, is caused by proteasomal degradation leading to a shorter half-life.

To understand whether Z-A1AT truncation variants lead to reduced polymer accumulation resulting from a loss of function from lower protein expression or gain of function, preventing onward Z-A1AT polymerization in a dominant-negative fashion, we transduced CHO cells with bicistronic vectors containing Z-A1AT, in addition to M-A1AT, Z-A1AT, Z-K367* or Z-E387*. As expected, CHO cells co-expressing Z-A1AT/Z-A1AT had significantly more inclusions than cells co-expressing Z-A1AT/M-A1AT (Fig. 4i). Cells expressing Z-K367* with Z-A1AT had similar levels of inclusions to Z-A1AT/M-A1AT-expressing cells. However, CHO cells expressing Z-E387* with Z-A1AT had significantly fewer ER inclusions than Z-A1AT/M-A1AT-expressing cells, suggesting that in addition to having reduced polymer load owing to reduced A1AT expression, expression of C-terminally truncated somatic variants might dominantly inhibit Z-A1AT polymerization. This will require further exploration, given that a dominant-negative function would have significant therapeutic implications.

## Discussion

Overall, we have identified convergent evolution of somatic variants in *SERPINA1* in the liver of patients with end-stage A1AT deficiency. The liver of each patient with PiZZ A1AT deficiency contained multiple hepatocyte clones with independent acquisition of variants in *SERPINA1*, the gene whose germline variant protein product drives the disease process. Remarkably, most of these somatic variants lead to truncation of the C-terminus of the A1AT protein and associate with reduced Z-A1AT polymer accumulation in vivo. This supports the idea that *SERPINA1* variants engender cellular advantage by mitigating Z-A1AT polymer accumulation^14,25^ and subsequent cellular stress. In vitro modeling of two of the C-terminal truncating variants has shown that these are associated with reduced Z-A1AT polymerization as well as reduced accumulation within and disruption of the ER.

**Extended Data Fig. 7:**
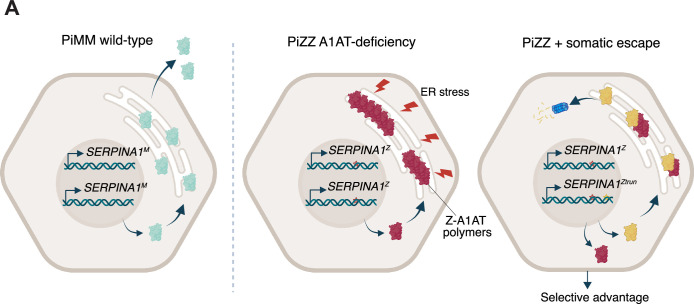
Model depicting cellular physiology of A1AT in differing genotypes. (**a**) Left: Homozygous MM-*SERPINA1* leads to expression, translation and secretion of functional A1AT protein; middle: Homozygous ZZ-*SERPINA1* leads to translation and subsequent polymerisation of Z-A1AT, leading to ER disruption. Right: Acquisition of a somatic escape variant in a single Z-*SERPINA1* allele leading to missense or truncation of the C-terminus reduces Z-A1AT polymer accumulation and ER disruption, providing a selective advantage compared to neighbouring hepatocytes with ZZ-*SERPINA1*.

Avoidance of A1AT polymerization and subsequent ER dysfunction would provide a significant selective advantage for hepatocyte clones in A1AT deficiency compared to neighboring Z-A1AT hepatocytes and therefore drive clonal expansion (Extended Data Fig. 7). This has important therapeutic implications in a disease that currently has no available therapies, aside from transplantation. Although therapies based on liver-directed RNA interference, suppressing the expression of Z-A1AT, have shown promise in mouse models^26^ and preliminary human trials^27^, targeted blockade or deletion of the C-terminus of A1AT may represent an alternative strategy for the suppression of polymer formation in A1AT deficiency. All of these strategies would promote hepatocyte survival and prevent progressive liver disease, but none would restore normal A1AT secretion or serum levels and therefore would probably not prevent progressive lung disease caused by A1AT deficiency.

The effect of disease-associated germline variants on subsequent somatic mutagenesis is relatively unexplored outside of hereditary cancer syndromes^28,29^. There are rare descriptions of somatic reversion mutations that correct germline variants in deleterious germline disorders: somatic genetic rescue has been seen in severe combined immunodeficiency, in which an individual with inherited biallelic *ADA* mutations acquired a reversion mutation, leading to outgrowth of a healthy lymphocyte population^30^. Somatic genetic rescue has been described in muscle tissue of patients with inherited Duchenne muscular dystrophy^31,32^ as well as patients with hereditary tyrosinemia, whereby somatic reversion mutations were identified in explanted liver tissue and their extent correlated with clinical severity^33^. Although similar to previous somatic genetic rescue, our identified variants in A1AT deficiency are not direct reversions but escape variants at a distinct site in the gene, mitigating the effect of a deleterious germline variant. Indirect somatic rescue mutations in distinct genes have previously been identified in some cases of Shwachman–Diamond syndrome, in which acquired variants in *EIF6* act to mitigate the deleterious effects of germline *EFL1* or *SBDS* mutations^34^.

Combined with our previous data in SLD, whereby distinct functional variants in metabolism genes were identified, these data suggest that differing disease processes will drive the expansion of clones containing disease-specific variants, providing a selective advantage in the face of specific microenvironmental pressures. Identification of the advantageous variants in different tissues, populations and disease states has the potential to reveal functional gene targets but also reveal specific domains within those targets that give insights into the disease mechanism and tractable therapeutic targets.

## Methods

### Liver samples

All liver samples were collected with written informed consent from Addenbrooke’s Hospital, Cambridge, UK, according to procedures approved by Local Research Ethics Committees (20/NI/0109, 16/NI/0196). All participants consented to publication of the research results. The liver samples were snap-frozen in liquid nitrogen and stored at −80 °C in the Human Research Tissue Bank of the Cambridge University Hospitals NHS Foundation Trust.

Background diseased liver tissue was obtained from individuals with A1AT deficiency or hemochromatosis who were undergoing liver transplantation for hepatocellular carcinoma or liver failure (Supplementary Table 1). Patients were identified from clinical history, pre-operative investigations and explant histology (Extended Data Fig. 1 and Supplementary Table 1). All patients with hemochromatosis had current or previously elevated serum ferritin with significant hepatocellular siderosis by Perls’ stain. Pre-operative clinical genotyping was only performed for the common C282Y and H63D variants of the *HFE* gene. Patients with A1AT deficiency were diagnosed based on PiZZ A1AT electrophoretic phenotype and sub-normal serum A1AT levels. The *SERPINA1*, *HFE*, *HJV*, *HAMP*, *TFR2*, *SLC40A1*, *BMP2* and *RAB6B* genotypes^3^ were derived from WGS or WES data (Extended Data Fig. 1 and Supplementary Table 2). Clinical and sequencing data from patients with SLD were derived from previous publications^5,11^.

The explant liver histology was reviewed by a specialist liver histopathologist (A.D., blinded to the other study results) and scored according to the Kleiner^35^ system on formalin-fixed paraffin-embedded samples away from the fresh–frozen block used for the LCM. The Kleiner score, developed for MASLD (to generate a cumulative MASLD activity score), was used in the absence of a validated scoring system for A1AT deficiency or hemochromatosis, allowing comparability between all study samples regardless of disease etiology. The histological findings were scored for siderosis by Perls’ staining using the Scheuer system^36^ and dPAS-positive globule deposition (Extended Data Fig. 1) as previously described^37^. Fibrosis was assessed using both the Kleiner^35^ and the Ishak^38^ scoring systems (Supplementary Table 1).

### Sample preparation

The protocols for preparing liver tissue sections, LCM and subsequent cell lysis, DNA extraction and WGS have been previously described^11,39^. In brief, for six biopsies (PD51605b, PD51606b, PD51607b, PD51608b, PD52285b and PD52286b), 20 µm-thick tissue sections (prepared with a Leica cryotome) were fixed with 70% ethanol. The other four biopsies (PD60232b, PD60233b, PD60802b and PD60803b) were fixed in PAXgene solutions (PreAnalytiX), processed using a Tissue Tek VIP 6 AI tissue processor (Sakura Finetek) and embedded in paraffin, and 16 µm-thick sections were generated using an Accu-Cut SRM 200 microtome (Sakura Finetek). All LCM sections were mounted on polyethylene naphthalene membrane glass slides (Leica Microsystems) and stained with H&E for subsequent LCM generation using a Leica Microsystems LMD 7000.

### LCM

For six biopsies (PD51605b, PD51606b, PD51607b, PD51608b, PD52285b and PD52286b), 48 microdissections were cut with a target area of 86,000 µm^2^, with the same *x*,*y*-region cut into the same well from two adjacent *z*-stacks. For the remaining four biopsies (PD60232b, PD60233b, PD60802b and PD60803b), cuts were taken at 166,000 µm^2^ without *z*-stacking. Overall, microdissection sizes corresponded to 800–1,000 hepatocytes. Images were taken before and after LCM. The microdissected samples were then lysed using the Arcturus PicoPure DNA Extraction Kit (Thermo Fisher Scientific) following the manufacturer’s instructions. DNA libraries for Illumina sequencing were prepared using a protocol optimized for low input amounts of DNA^39^. The resulting libraries were submitted for paired-end WGS or WES.

### Exome sequencing

Exome capture was performed using either SureSelect All Exon v5 bait set (Agilent, S04380110) or Twist Human Core Exome (Twist Biosciences) bait set. Samples were multiplexed and sequenced using 150 bp paired-end reads on an Illumina NovaSeq 6000 (average pool size of 40). Paired-end reads were aligned to human genome assembly GRCh38 using BWA-MEM^40^. Duplicate reads were marked using biobambam^41^ and sample contamination estimates were calculated using VerifyBamID^42^. Library complexity and coverage statistics were calculated using Picard (http://broadinstitute.github.io/picard). The mean on-target coverage across all samples and genes was 48×. Across donors, median coverage ranged from 39× (PD52285b) to 65× (PD60233b).

### WGS

WGS was performed using 150 bp paired-end reads on an Illumina NovaSeq 6000. DNA sequences were aligned to the GRCh38 reference genome using BWA-MEM. Duplicate reads were marked using biobambam^41^. The calculation of library statistics was performed using the CollectWgsMetrics function of Picard Tools, rather than the CollectHsMetrics function used for exome sequencing data. The mean coverage across all samples was 42×.

### Calling of single nucleotide variants and indels from exome and WGS data

Single nucleotide variants (SNVs) were called using the CaVEMan (cancer variants through expectation maximization) algorithm^43^ against an in silico unmatched normal: a BAM file generated from the human reference genome (GRCh38). Indel calling was performed using cgpPindel^44^, with filtering strategies the same as for SNVs. In addition to the default CaVEMan filters, putative SNVs were required to have a median alignment score of reads showing the variant allele of at least 0.87 and fewer than half supporting reads being clipped (median number of soft-clipped bases, CLPM = 0). Duplicate reads and LCM library preparation-specific artifactual variants resulting from the incorrect processing of secondary cruciform DNA structures were removed with bespoke post-processing filtering^39^. Across all samples from the same patient, we force-called the SNVs and indels that were called in any sample, using a cutoff for read mapping quality (30) and base quality (25). To filter shared artifacts, we applied a beta-binomial-based filtering approach as previously described^45^. Mutations with a dispersion estimate of ≥0.1 were considered true somatic variants. Putative germline variants were filtered using a one-sided exact binomial test used on the aggregated counts of reads supporting the variant and the total depth at that site, as described previously^45^. This tests whether the observed variant counts are likely to have come from a germline distribution or from a distribution with a lower true VAF (probably somatic). For sex chromosomes in male individuals, the binomial probability (true VAF) for comparison was set to 0.95 rather than 0.5. The resulting *P* values were corrected for multiple testing using a Benjamini–Hochberg correction. Any variant with a *q*-value of ≤10^−5^ was classified as a putative somatic variant.

### SV calling from WGS data

SVs were called with GRIDDS^46^ (v.2.9.4), used with default settings. SVs larger than 1 kb in size with QUAL ≥ 250 were included. For SVs smaller than 30 kb, only SVs with QUAL ≥ 300 were included. Furthermore, SVs that had assemblies from both sides of the breakpoint were only considered if they were supported by at least four discordant and two split reads. SVs with imprecise breakends (that is, the distance between the start and end positions of >10 bp) were filtered out. We further filtered out SVs for which the standard deviation of the alignment positions at either ends of the discordant read pairs was smaller than five. To remove potential germline SVs and artifacts, we generated the panel of normals by adding in-house normal samples (*n* = 350) to the GRIDSS panel of normals. SVs found in at least three different samples in the panel of normals or in matched normal tissues were removed.

### Copy number variant calling from WGS data

Somatic copy number variants were called using the Allele-Specific Copy number Analysis of Tumors (ASCAT) algorithm^47^ as part of the ascatNGS package^48^ (https://github.com/Crick-CancerGenomics/ascat). ASCAT was run with default parameters with the exception of a segmentation penalty of 100. A bespoke filtering algorithm, ascatPCA, was used to reduce the number of false-positive calls that can arise when analyzing genome sequences from normal tissue (https://github.com/hj6-sanger/ascatPCA). ascatPCA extracts a noise profile by aggregating the logR ratio from across a panel of normal unrelated samples and subtracts this signature from that observed in the sample being analyzed using principal component analysis.

### Extraction of mutational signatures

As previously described^5,11^, the hierarchical Dirichlet process (HDP) algorithm, as implemented in the HDP R package (https://github.com/nicolaroberts/hdp), was used to extract mutational signatures based on a reference catalog of 65 previously identified 192-context-based mutational signatures from the Pan-Cancer Analysis of Whole Genomes (PCAWG) study^49^ as well as a novel liver signature identified previously in CLD^5^.

### Bayesian Dirichlet process for clustering VAFs across multiple samples

The nonparametric Bayesian HDP was used to group SNVs based on similar VAFs identified across multiple microdissections in each liver sample. This method, called *N*-dimensional Dirichlet process (NDP) clustering, has been detailed previously^11^. We ran the algorithm with 15,000 burn-in iterations, followed by 25,000 iterations of posterior Gibbs sampling for the clustering process. During each iteration, there is a defined probability that mutations will be allocated to new clusters that did not exist in the previous iteration. Existing clusters can also be eliminated if member mutations are reassigned to another cluster. This adaptive process allows for the dynamic adjustment of the number of clusters throughout the sampling. To prevent the formation of uninformative clusters, we capped the number of SNV clusters at 100 per patient. We also used a multi-threaded version of the ECR algorithm, adapted from the label.switching R package (10.18637/jss.v069.c01), to correct for label switching efficiently. For subsequent reconstruction of phylogenetic trees, we only considered SNV clusters that included at least 50 distinct mutations.

### Construction of phylogenetic trees

The statistical pigeonhole principle^50^ was used to deduce the phylogenetic clonal relationships among SNV clusters identified in each patient by the NDP algorithm. Here, each cluster forms a branch on a phylogenetic tree. Evidence that a cluster is considered nested within another is considered strong if its mutation-carrying cell fraction is consistently lower than that of another cluster across all sampled microdissections, and if the combined mutant cell fractions exceed 100%. A combined cell fraction of ≤100% indicates only weak evidence of such nesting. If only certain microdissections show a lower cell fraction for one SNV cluster compared to another, these clusters are deemed independent, not nested within one another. Our analysis was restricted to clusters with a mutant cell fraction of >0.05. We calculated the cell fraction for each SNV cluster by doubling the median VAF in each microdissection, assuming diploidy. SNV clusters with microdissections lacking shared mutations with others in the same cluster were divided into new, independent clusters. These were then reassessed for their phylogenetic relationships to all other clusters from the same patient biopsy, using the pigeonhole principle. Additionally, a naive Bayes algorithm was used to categorize each identified indel into the SNV clusters detected by the NDP algorithm.

### Analysis of driver variants

To determine whether any coding variants were under selection in diseased liver tissues, the dN/dScv method^12^ on the gene level was used. This algorithm identifies genes with an excess of non-synonymous mutations relative to the expected number from the synonymous mutation rate. For this analysis, variants called from whole genomes were collapsed to unique events per SNV cluster identified by the NDP algorithm. Any variants identified in exome data that were not already called in whole genomes from the same patient were collapsed to unique mutations per individual and added to the dN/dScv input. In addition, we conservatively collapsed SNVs separated by ≤10 bp within the same LCM sample into multinucleotide variants. Genes with *q* values of <0.1 were considered to be under positive selection.

### Extraction of mutational signatures from SNV contexts using HDP

To identify possibly undiscovered mutational signatures in the liver from A1AT deficiency and hemochromatosis patients, the HDP (https://github.com/nicolaroberts/hdp) was run on the 96 trinucleotide counts of all microdissected samples, collapsed to unique mutations across samples. To avoid over-fitting, samples with fewer than 50 mutations were not included in the signature extraction. HDP was run with individual patients as the hierarchy, in 20 independent chains, with a burn-in of 20,000 and the collection of 100 posterior samples off each chain with 200 iterations between each. Owing to the lack of novel signatures in this data set, the remainder of the mutational signature analysis was performed by fitting the identified set of signatures from HDP to trinucleotide counts from each microdissection using the R package sigfit (10.1101/372896).

### Protein structure prediction modeling

Structural predictions of the Z-K367*, Z-E387* and Z-M398delFS somatic mutant A1AT proteins were performed using Pymol (v.2.5.2, Schrodinger) to map amino acid sequences onto the crystal structure of native human A1AT (PDB 1QLP)^15^, identifying secondary structures lost in somatic mutant proteins. To predict the ability of somatic mutant proteins to form mixed polymers with full-length Z-A1AT, their sequences were mapped onto a single protomer within the crystal structure of a human Z-A1AT polymer (PDB 3T1P)^16^. Atoms of the 3T1P protomer that are lost through truncation of somatic mutants were removed from the displayed image to highlight any incompatibility with polymerization. The two missense residues in Z-M398delFS (F396Y, L397D) were modeled using the Pymol mutagenesis function, selecting side chain rotamers of Y396 and D397 that best conserved the resolved density in 3T1P. Somatic mutant structural models did not undergo energy minimization or geometry optimization processes and hence are used only to predict the feasibility of polymerization, not changes to the protein fold. Structural prediction of Z-M398delFS A1AT was carried out using AlphaFold 3 (ref. ^51^), in which the top-ranked model is displayed.

### Cloning and constructs

Somatic variants of Z-A1AT (Z-K367*, Z-E387*) were generated by site-directed mutagenesis of the Z-A1AT coding sequence (untagged and N-terminally tagged with HaloTag) used previously^17^. The sequences of primers used in this study are provided in Supplementary Table 3. Bicistronic vectors for co-expression of A1AT with an ER marker protein were generated from pGL4.2 encoding A1AT tagged with HaloTag (M-A1AT, Z-A1AT, Z-K367*-A1AT and Z-E387*-A1AT variants) and the ER marker protein moxsynGFP-KDEL by Gibson assembly (New England Biolabs) to form pGL4.2_HaloTag-A1AT_ER-moxGFP-KDEL variants. For the expression of two A1AT forms to mimic heterozygosity, the ER-moxGFP-KDEL in pGL4.2_HaloTag-Z-A1AT_ER-moxGFP-KDEL (SJM1134) was replaced with untagged A1AT variant coding sequences using Gibson assembly. Sequencing of the plasmids was carried out at Plasmidsaurus (Oxford Nanopore Technologies).

### Mammalian cell culture

CHO cells (Clontech) were cultured in F12 Ham’s nutrient mixture (Merck) supplemented with 10% FBS and 2 mM GlutaMAX (Thermo Fisher Scientific) at 37 °C and 5% CO_2_. CHO Tet-ON cells with inducible expression of Z-A1AT were cultured in high-glucose DMEM (Merck) supplemented with 10% tetracycline-free FBS, 2 mM GlutaMAX and non-essential amino acids (Thermo Fisher Scientific). COS7 cells (MERCK) were cultured in high-glucose DMEM supplemented with 10% FBS and 2 mM GlutaMAX.

### Live-cell imaging

CHO parental cells were transfected with a bicistronic vector encoding moxGFP-KDEL and HaloTag-A1AT (M, Z, Z-K367* and Z-E387* variants), performed according to the Lipofectamine LTX reagent protocol (Life Technology). Then, 24 h later, cells were sorted for a GFP-positive population using the BD Influx Cell Sorter and then seeded at 1 × 10^4^ cells per cm^2^ in an eight-well ibidi slide. Imaging was performed 48 h after seeding. Before imaging, cells were labeled with 0.5 µM JFX549 (Janelia Fluor) in Opti-MEM for 15 min at 37 °C. Airyscan images were collected on an LSM880 (Zeiss) confocal microscope using an Airyscan detector and processed using the Zen (v.2.6) software package (Black edition, Zeiss). Quantitation of ER morphology using co-expression of ER-moxGFP-KDEL was performed using standard confocal imaging using the ImageJ (Fiji, v.2.14.0/1.54f) software package. Cells were counted based on ER morphology and categorized as either reticular ER or as containing ER inclusions. Quantitation was performed using the moxGFP-KDEL channel to avoid bias introduced by variable accumulation (and hence fluorescence intensity) of different A1AT variants. To quantify ER morphology in cells co-expressing variants of untagged antitrypsin with HaloTagged Z-A1AT, as a mimic of heterozygous expression, CHO cells were transfected as described above with the relevant bicistronic vector. Cells were labeled with JFX549 HaloTag ligand after 48 h expression, then imaged using a Zeiss LSM980 in Airyscan mode. Images were processed using Zen blue software before a gamma correction of 0.5 was applied to allow simultaneous visualization of high-intensity and low-intensity features. Cells were then categorized as having either reticular or inclusion-containing ER morphology.

### Single-particle tracking

Single-particle tracking of HaloTag-A1AT variants was performed as described previously^18^ using Trackmate (v.7) software^22^. In brief, COS7 cells were transfected 4 h after seeding using 1 µg DNA and 3 µl Fugene 6 (Promega) as per the manufacturer’s instructions, to drive expression of an ER marker protein (mEmerald-KDEL) and HaloTag-A1AT variants. Cells were imaged 18 h after transfection, following labeling with PA-JF646 photoactivatable ligand^52^.

### Sandwich ELISA for A1AT

CHO cells were seeded at a density of 2.08 × 10^4^ cells per cm^2^ in six-well cell culture plates (Greiner Bio-One), as described above for CHO parental cells. Cell culture media was substituted with 1 ml of Opti-MEM reduced serum medium (ThermoFisher Scientific) 24 h after transfection. Conditioned Opti-MEM and cell lysates were collected 48 h after transfection and subject to ELISA for A1AT as described previously^17^, using the monoclonal anti-A1AT antibody mAb_3C11_ (ref. ^53^) (a kind gift from David Lomas).

### Native PAGE and SDS–PAGE

Native PAGE and SDS–PAGE were performed as described previously^18^. In brief, parental CHO and Tet-On CHO cells were seeded at a density of 2.08 × 10^4^ cells per cm^2^ and transfected as described above for CHO parental cells. For native PAGE, sonicated soluble lysate containing 80 μg of protein, or an equivalent volume of the corresponding insoluble lysate, was loaded onto an acrylamide native gel (resolving gel composition: 7.5% w/v acrylamide–bisacrylamide mixture (37.5:1), 0.37 mM Tris-HCl pH 8.8, 0.12% w/v APS, 0.2% v/v TEMED; stacking gel composition: 5.3% w/v acrylamide–bisacrylamide mixture (37.5:1), 110 mM Tris-HCl pH 6.8, 0.125% w/v APS, 0.15% v/v TEMED). For SDS–PAGE, soluble lysate containing 80 μg of protein, or an equivalent volume of the corresponding insoluble lysate, was loaded on a 10% acrylamide gel. A monoclonal α_1_-antitrypsin antibody (MA5-15521, Thermo Fisher Scientific) raised against a peptide consisting of amino acids 40–184 of human A1AT was used for SDS–PAGE immunoblotting. A polyclonal antibody raised against full-length human A1AT (A0409, Sigma-Aldrich) was used for native PAGE western blot (total α_1_-antitrypsin pool). The human A1AT polymer-specific mAb_2C1_ antibody (HM2289, Hycult Biotechnology) was used for native PAGE detection of polymers.

### Z-A1AT polymer immunohistochemistry

Immunohistochemistry staining was performed on liver tissue sections from subjects with A1AT deficiency, adjacent to those on which LCM was previously performed. Sections (5 µm thick) were cut from PAXgene-fixed paraffin-embedded tissue blocks and mounted on Superfrost Plus glass microscope slides. Sections were dewaxed by sequential immersion in xylene two times for 2 min, 100% ethanol two times for 2 min, 70% ethanol for 1 min and then deionized water for 1 min. Endogenous peroxidase was blocked by soaking slides in methanol with 0.3% hydrogen peroxide solution for 30 min. After washing the slides in TBS, off-target secondary antibody was blocked by soaking the tissue sections in 3% normal horse serum (VECTASTAIN Elite ABC-HRP Mouse IgG Kit, Vector Laboratories) in TBS for 1 h. Tissue sections were washed in TBS and incubated overnight in 1:50 dilution of the polymer-specific mAb_2C1_ antibody (Hycult Biotech, cat. no. HM2289). The next day, tissue sections were incubated with horseradish peroxidase (HRP)-conjugated horse anti-mouse IgG secondary antibody (1:200, VECTASTAIN Elite ABC-HRP Mouse IgG Kit, Vector Laboratories) for 1 h. After the removal of unbound antibodies, HRP activity was developed with diaminobenzene. The sections were then counterstained with Mayer’s hematoxylin, rinsed, rehydrated, mounted and imaged using a NanoZoomer 2.0-HT slide scanner (Hamamatsu Photonics).

The 2C1 staining intensity from regions of interest was scored (0–3) by four independent scorers (V.K., J.C., S.J.M. and T.C.) who were blinded to the genotype of the region of interest; a mean of these scores was reported. Regions of interest were grouped based on *SERPINA1* genotype: non-truncating variants included L12delFS, F47InsFS, L124delFS, L144delFS, Q180delFS, K192insFS, V205delFS, Q236delFS and L397delIF. Truncating variants included K367*, G373delFS, G373insFS, M382delFS, S383insFS, P386insFS, E387insFS, E387* and F396delFS.

### HaloTag pulse–chase of A1AT

CHO parental cells were transfected using the Invitrogen Neon Transfection System with 5 μg of DNA encoding HaloTag-A1AT (M, Z, Z-K367* and Z-E387* variants) per 5 × 10^5^ cells using three electric pulses at 1,400 V and 10 ms width. Cells were labeled with PA-JF646 photoactivatable ligand after 42 h and chased for a further 24 h. Cells were collected at 0, 1.5, 3, 6, 12 and 24 h time points. Cell lysates were sonicated in a water bath sonicator for 30 min at 4 °C (whole-cell lysate). Then, 60 μg of protein lysates from the first time point and an equivalent volume of protein lysates from subsequent time points were prepared in SDS loading buffer for SDS–PAGE analysis. Gel fluorescence was detected using a Li-Cor CLx scanner.

### HaloTag pulse–chase of Z-K367* with inhibitors of degradation

CHO parental cells were transfected using the Invitrogen Neon Transfection System as above. Cells were labeled with PA-JF650 photoactivatable ligand after 42 h and substituted with media containing 5 μM lactacystin, 100 nM bafilomycin or both and chased for a further 6 h. Cells were collected at 0, 1.5, 3 and 6 h. Then, 50 μg of protein lysates from the first time point and equivalent volumes of protein lysates from subsequent time points were prepared in SDS loading buffer for SDS–PAGE analysis as previously described. Gel fluorescence was detected using a Li-Cor CLx scanner.

### HaloLink pulldown of A1AT

CHO Tet-ON cells with inducible expression of untagged Z-A1AT, as described above, were treated with 1 μg ml^−1^ doxycycline for 48 h. Cells were collected by washing twice with PBS on ice before applying 200 µl of ice-cold lysis buffer (50 mM Tris-HCl pH 7.4, 150 mM NaCl, 1% Triton) containing 1× protease inhibitor (G6521, Promega). The cell lysate supernatant was used for the subsequent steps of the HaloLink pulldown analysis. Equal amounts of protein lysates were incubated with Magne HaloTag beads (Promega, cat. no. G7281) overnight at 4 °C in a rotor. Beads were washed four times with lysis buffer, and protein interactors of HaloTag fusion proteins were liberated with 2× SDS–PAGE sample buffer (312.5 mM Tris-HCl pH 6.8, 50% v/v glycerol, 10% w/v SDS, 0.05% w/v Bromophenol Blue, 50 mM dithiothreitol) by heating at 75 °C for 10 min and separation from HaloTag bead. SDS–PAGE was performed on eluted material and 80 μg of input, followed by immunoblotting as described above.

### Statistics and reproducibility

Statistical analyses were performed using R (v.4.3.1) or GraphPad Prism (v.9); statistical tests used, numbers of biological replicates and the *P* values are described in the figure legends; *q* values were calculated using the Benjamini–Hochberg correction for multiple hypothesis testing. No data were excluded from the analyses. The experiments were not randomized and the investigators, apart from the pathologist, were not blinded to allocation during the experiments and outcome assessment.

### Reporting summary

Further information on research design is available in the Nature Portfolio Reporting Summary linked to this article.

## Online content

Any methods, additional references, Nature Portfolio reporting summaries, source data, extended data, supplementary information, acknowledgements, peer review information; details of author contributions and competing interests; and statements of data and code availability are available at 10.1038/s41588-025-02125-1.

## Supplementary information

Supplementary Information Supplementary Tables 1–3.

Reporting Summary

## Source data

Source Data Fig. 1 Supplementary figure: full scans of immunoblots used in the indicated figures, including molecular-weight markers.

## Data Availability

WGS and WES data have been deposited in the European Genome–phenome Archive (EGA) (https://ega-archive.org). WGS data have been deposited with EGA accession number EGAD00001015430 and exome sequencing data have been deposited with accession number EGAD00001015431. Existing DNA-sequencing datasets from the liver of subjects with SLD used in the study are deposited in EGA with accession number EGAD00001006255. The crystal structure of native human A1AT was obtained from the RCSB Protein Data Bank (https://www.rcsb.org/structure/1qlp). Source data are provided with this paper.

## Code availability

Previously generated code that was used in this work was obtained from GitHub: single-nucleotide substitutions were called using the CaVEMan algorithm (v.1.15.1) (https://github.com/cancerit/CaVEMan); small insertions and deletions were called using the Pindel algorithm (v.3.7.0) (https://github.com/cancerit/cgpPindel); selection analysis was performed using the dNdScv package (v.0.0.1.0) (https://github.com/im3sanger/dndscv). Newly generated code underpinning the downstream analyses is also available on GitHub (https://github.com/nataliabrz/positive_selection_in_A1AT_deficiency) and the specific versions (v.1.0.0) are captured on Zenodo^54^. Whole-genome and whole-exome somatic mutation calls necessary to reproduce the analyses in the GitHub repository are available on Mendeley Data (https://data.mendeley.com/datasets/vhybvj2g9p/1)^55^.

## References

[1] Huang, D. Q. et al. Global epidemiology of cirrhosis—aetiology, trends and predictions. *Nat. Rev. Gastroenterol. Hepatol.* **20**, 388–398 (2023). 36977794 10.1038/s41575-023-00759-2 PMC10043867

[2] Berg, N. O. & Eriksson, S. Liver disease in adults with alpha1-antitrypsin deficiency. *N. Engl. J. Med.* **287**, 1264–1267 (1972). 4117996 10.1056/NEJM197212212872502

[3] Girelli, D. et al. Hemochromatosis classification: update and recommendations by the BIOIRON Society. *Blood* **139**, 3018–3029 (2021). 10.1182/blood.2021011338 PMC11022970 34601591

[4] Carrell, R. W. & Lomas, D. A. Alpha1-antitrypsin deficiency—a model for conformational diseases. *N. Engl. J. Med.* **346**, 45–53 (2002). 11778003 10.1056/NEJMra010772

[5] Ng, S. W. K. et al. Convergent somatic mutations in metabolism genes in chronic liver disease. *Nature* **598**, 473–478 (2021). 34646017 10.1038/s41586-021-03974-6

[6] Wang, Z. et al. Positive selection of somatically mutated clones identifies adaptive pathways in metabolic liver disease. *Cell* **186**, 1968–1984.e20 (2023). 37040760 10.1016/j.cell.2023.03.014 PMC10321862

[7] Jamialahmadi, O. et al. Exome-wide association study on alanine aminotransferase identifies sequence variants in the GPAM and APOE associated with fatty liver disease. *Gastroenterology* **160**, 1634–1646.e7 (2021). 33347879 10.1053/j.gastro.2020.12.023

[8] Ghouse, J. et al. Integrative common and rare variant analyses provide insights into the genetic architecture of liver cirrhosis. *Nat. Genet.* **56**, 827–837 (2024). 38632349 10.1038/s41588-024-01720-y PMC11096111

[9] Verweij, N. et al. Germline mutations in CIDEB and protection against liver disease. *New Engl. J. Med.* **387**, 332–344 (2022). 35939579 10.1056/NEJMoa2117872

[10] Zhu, M. et al. Somatic mutations increase hepatic clonal fitness and regeneration in chronic liver disease. *Cell* **177**, 608–621.e12 (2019). 30955891 10.1016/j.cell.2019.03.026 PMC6519461

[11] Brunner, S. F. et al. Somatic mutations and clonal dynamics in healthy and cirrhotic human liver. *Nature* **574**, 538–542 (2019). 31645727 10.1038/s41586-019-1670-9 PMC6837891

[12] Martincorena, I. et al. Universal patterns of selection in cancer and somatic tissues. *Cell* **171**, 1029–1041.e21 (2017). 29056346 10.1016/j.cell.2017.09.042 PMC5720395

[13] Ally, A. et al. Comprehensive and integrative genomic characterization of hepatocellular carcinoma. *Cell* **169**, 1327–1341.e23 (2017). 28622513 10.1016/j.cell.2017.05.046 PMC5680778

[14] Faull, S. V. et al. The structural basis for Z α_1_-antitrypsin polymerization in the liver. *Sci. Adv.* **6**, eabc1370 (2020). 33087346 10.1126/sciadv.abc1370 PMC7577719

[15] Elliott, P. R., Pei, X. Y., Dafforn, T. R. & Lomas, D. A. Topography of a 2.0 Å structure of α_1_‐antitrypsin reveals targets for rational drug design to prevent conformational disease. *Protein Sci.* **9**, 1274–1281 (2000). 10933492 10.1110/ps.9.7.1274 PMC2144685

[16] Yamasaki, M., Sendall, T. J., Pearce, M. C., Whisstock, J. C. & Huntington, J. A. Molecular basis of α_1_‐antitrypsin deficiency revealed by the structure of a domain‐swapped trimer. *EMBO Rep.* **12**, 1011–1017 (2011). 21909074 10.1038/embor.2011.171 PMC3185345

[17] Dickens, J. A. et al. The endoplasmic reticulum remains functionally connected by vesicular transport after its fragmentation in cells expressing Z‐α_1_‐antitrypsin. *FASEB J.* **30**, 4083–4097 (2016). 27601439 10.1096/fj.201600430R PMC5102109

[18] Chambers, J. E. et al. Z-α_1_-antitrypsin polymers impose molecular filtration in the endoplasmic reticulum after undergoing phase transition to a solid state. *Sci. Adv.* **8**, eabm2094 (2022). 35394846 10.1126/sciadv.abm2094 PMC8993113

[19] Segeritz, C.-P. et al. hiPSC hepatocyte model demonstrates the role of unfolded protein response and inflammatory networks in α_1_-antitrypsin deficiency. *J. Hepatol.* **69**, 851–860 (2018). 29879455 10.1016/j.jhep.2018.05.028 PMC6562205

[20] Ordóñez, A. et al. Endoplasmic reticulum polymers impair luminal protein mobility and sensitize to cellular stress in alpha_1_‐antitrypsin deficiency. *Hepatology* **57**, 2049–2060 (2013). 23197448 10.1002/hep.26173 PMC3871212

[21] Grimm, J. B. et al. A general method to improve fluorophores for live-cell and single-molecule microscopy. *Nat. Methods* **12**, 244–250 (2015). 25599551 10.1038/nmeth.3256 PMC4344395

[22] Tinevez, J.-Y. et al. TrackMate: an open and extensible platform for single-particle tracking. *Methods* **115**, 80–90 (2017). 27713081 10.1016/j.ymeth.2016.09.016

[23] Ronzoni, R. et al. The molecular species responsible for α_1_‐antitrypsin deficiency are suppressed by a small molecule chaperone. *FEBS J.* **288**, 2222–2237 (2021). 33058391 10.1111/febs.15597 PMC8436759

[24] Lieberman, J., Mittman, C. & Gordon, H. W. Alpha_1_ antitrypsin in the livers of patients with emphysema. *Science* **175**, 63–65 (1972). 4109412 10.1126/science.175.4017.63

[25] Behrens, M. A. et al. The shapes of Z-α_1_-antitrypsin polymers in solution support the C-terminal domain-swap mechanism of polymerization. *Biophys. J.* **107**, 1905–1912 (2014). 25418171 10.1016/j.bpj.2014.08.030 PMC4213723

[26] Wooddell, C. I. et al. Development of an RNAi therapeutic for alpha-1-antitrypsin liver disease. *JCI Insight* **5**, e135348 (2020). 32379724 10.1172/jci.insight.135348 PMC7406265

[27] Strnad, P. et al. Fazirsiran for liver disease associated with alpha1-antitrypsin deficiency. *N. Engl. J. Med.* **387**, 514–524 (2022). 35748699 10.1056/NEJMoa2205416

[28] Robinson, P. S. et al. Increased somatic mutation burdens in normal human cells due to defective DNA polymerases. *Nat. Genet.* **53**, 1434–1442 (2021). 34594041 10.1038/s41588-021-00930-y PMC8492474

[29] Lee, B. C. H. et al. Mutational landscape of normal epithelial cells in Lynch Syndrome patients. *Nat. Commun.* **13**, 2710 (2022). 35581206 10.1038/s41467-022-29920-2 PMC9114395

[30] Hirschhorn, R. et al. Spontaneous in vivo reversion to normal of an inherited mutation in a patient with adenosine deaminase deficiency. *Nat. Genet.* **13**, 290–295 (1996). 8673127 10.1038/ng0796-290

[31] Burrow, K. L. et al. Dystrophin expression and somatic reversion in prednisone‐treated and untreated Duchenne dystrophy. *Neurology* **41**, 661–666 (1991). 1781820 10.1212/wnl.41.5.661

[32] Klein, C. J. et al. Somatic reversion/suppression in Duchenne muscular dystrophy (DMD): evidence supporting a frame-restoring mechanism in rare dystrophin-positive fibers. *Am. J. Hum. Genet.* **50**, 950–959 (1992). 1570844 PMC1682584

[33] Demers, S. I., Russo, P., Lettre, F. & Tanguay, R. M. Frequent mutation reversion inversely correlates with clinical severity in a genetic liver disease, hereditary tyrosinemia. *Hum. Pathol.* **34**, 1313–1320 (2003). 14691918 10.1016/s0046-8177(03)00406-4

[34] Tan, S. et al. Somatic genetic rescue of a germline ribosome assembly defect. *Nat. Commun.* **12**, 5044 (2021). 34413298 10.1038/s41467-021-24999-5 PMC8377010

[35] Kleiner, D. E. et al. Design and validation of a histological scoring system for nonalcoholic fatty liver disease. *Hepatology* **41**, 1313–1321 (2005). 15915461 10.1002/hep.20701

[36] Scheuer, P. J., Williams, R. & Muir, A. R. Hepatic pathology in relatives of patients with haemochromatosis. *J. Pathol. Bacteriol.* **84**, 53–64 (1962). 14498313

[37] Dawwas, M. F., Davies, S. E., Griffiths, W. J. H., Lomas, D. A. & Alexander, G. J. Prevalence and risk factors for liver involvement in individuals with PiZZ-related lung disease. *Am. J. Respir. Crit. Care Med.* **187**, 502–508 (2013). 23262512 10.1164/rccm.201204-0739OC

[38] Ishak, K. et al. Histological grading and staging of chronic hepatitis. *J. Hepatol.* **22**, 696–699 (1995). 7560864 10.1016/0168-8278(95)80226-6

[39] Ellis, P. et al. Reliable detection of somatic mutations in solid tissues by laser-capture microdissection and low-input DNA sequencing. *Nat. Protoc.* **16**, 841–871 (2021). 33318691 10.1038/s41596-020-00437-6

[40] Li, H. & Durbin, R. Fast and accurate short read alignment with Burrows–Wheeler transform. *Bioinformatics* **25**, 1754–1760 (2009). 19451168 10.1093/bioinformatics/btp324 PMC2705234

[41] Tischler, G. & Leonard, S. biobambam: tools for read pair collation based algorithms on BAM files. *Source Code Biol. Med.* **9**, 13 (2014).

[42] Jun, G. et al. Detecting and estimating contamination of human DNA samples in sequencing and array-based genotype data. *Am. J. Hum. Genet.* **91**, 839–848 (2012). 23103226 10.1016/j.ajhg.2012.09.004 PMC3487130

[43] Jones, D. et al. cgpCaVEManWrapper: simple execution of CaVEMan in order to detect somatic single nucleotide variants in NGS data. *Curr. Protoc. Bioinformatics* **56**, 15.10.1–15.10.18 (2016). 27930805 10.1002/cpbi.20 PMC6097605

[44] Raine, K. M. et al. cgpPindel: identifying somatically acquired insertion and deletion events from paired end sequencing. *Curr. Protoc. Bioinformatics* **52**, 15.7.1–15.7.12 (2015). 26678382 10.1002/0471250953.bi1507s52 PMC6097606

[45] Coorens, T. H. H. et al. Extensive phylogenies of human development inferred from somatic mutations. *Nature* **597**, 387–392 (2021). 34433963 10.1038/s41586-021-03790-y

[46] Cameron, D. L. et al. GRIDSS: sensitive and specific genomic rearrangement detection using positional de Bruijn graph assembly. *Genome Res.* **27**, 2050–2060 (2017). 29097403 10.1101/gr.222109.117 PMC5741059

[47] Loo, P. V. et al. Allele-specific copy number analysis of tumors. *Proc. Natl Acad. Sci. USA* **107**, 16910–16915 (2010). 20837533 10.1073/pnas.1009843107 PMC2947907

[48] Raine, K. M. et al. ascatNgs: identifying somatically acquired copy‐number alterations from whole‐genome sequencing data. *Curr. Protoc. Bioinformatics* **56**, 15.9.1–15.9.17 (2016). 27930809 10.1002/cpbi.17 PMC6097604

[49] Alexandrov, L. B. et al. The repertoire of mutational signatures in human cancer. *Nature* **578**, 94–101 (2020). 32025018 10.1038/s41586-020-1943-3 PMC7054213

[50] Nik-Zainal, S. et al. The life history of 21 breast cancers. *Cell* **149**, 994–1007 (2012). 22608083 10.1016/j.cell.2012.04.023 PMC3428864

[51] Abramson, J. et al. Accurate structure prediction of biomolecular interactions with AlphaFold 3. *Nature* **630**, 493–500 (2024). 38718835 10.1038/s41586-024-07487-w PMC11168924

[52] Grimm, J. B. et al. Bright photoactivatable fluorophores for single-molecule imaging. *Nat. Methods* **13**, 985–988 (2016). 27776112 10.1038/nmeth.4034

[53] Tan, L. et al. Characterising the association of latency with α_1_-antitrypsin polymerisation using a novel monoclonal antibody. *Int. J. Biochem. Cell Biol.* **58**, 81–91 (2015). 25462157 10.1016/j.biocel.2014.11.005 PMC4305080

[54] Brzozowska, N. Selection for somatic escape variants in SERPINA1 in the liver of patients with alpha-1 anti-trypsin deficiency. *Zenodo*10.5281/zenodo.14771943 (2024). 10.1038/s41588-025-02125-1 PMC11985350 40065168

[55] Brzozowska, N. Selection for somatic escape variants in SERPINA1 in the liver of patients with alpha-1 anti-trypsin deficiency. *Mendeley Data*10.17632/vhybvj2g9p.1 (2024).

