# Supplementary material for: Selection for somatic escape variants in SERPINA1 in the liver of patients with alpha-1 antitrypsin deficiency
Source: Nat Genet. 2025 Mar 10;57(4):875–83. doi: 10.1038/s41588-025-02125-1 (PMC11985350; doi:10.1038/s41588-025-02125-1)
Supplement: Supplementary file 2 — Reporting Summary [file 41588_2025_2125_MOESM2_ESM.pdf]

nature portfolio

Corresponding author(s): Matthew Hoare

Last updated by author(s): Jan 30, 2025

Reporting Summary

Nature Portfolio wishes to improve the reproducibility of the work that we publish. This form provides structure for consistency and transparency in reporting. For further information on Nature Portfolio policies, see our Editorial Policies and the Editorial Policy Checklist.

Statistics

For all statistical analyses, confirm that the following items are present in the figure legend, table legend, main text, or Methods section.

n/a	Confirmed	
☐	☒	The exact sample size (*n*) for each experimental group/condition, given as a discrete number and unit of measurement
☐	☒	A statement on whether measurements were taken from distinct samples or whether the same sample was measured repeatedly
☐	☒	The statistical test(s) used AND whether they are one- or two-sided<br>*Only common tests should be described solely by name; describe more complex techniques in the Methods section.*
☐	☒	A description of all covariates tested
☐	☒	A description of any assumptions or corrections, such as tests of normality and adjustment for multiple comparisons
☐	☒	A full description of the statistical parameters including central tendency (e.g. means) or other basic estimates (e.g. regression coefficient) AND variation (e.g. standard deviation) or associated estimates of uncertainty (e.g. confidence intervals)
☐	☒	For null hypothesis testing, the test statistic (e.g. *F*, *t*, *r*) with confidence intervals, effect sizes, degrees of freedom and *P* value noted<br>*Give P values as exact values whenever suitable.*
☒	☐	For Bayesian analysis, information on the choice of priors and Markov chain Monte Carlo settings
☐	☒	For hierarchical and complex designs, identification of the appropriate level for tests and full reporting of outcomes
☒	☐	Estimates of effect sizes (e.g. Cohen's *d*, Pearson's *r*), indicating how they were calculated

Our web collection on statistics for biologists contains articles on many of the points above.

Software and code

Policy information about availability of computer code

Data collection	No software was used
Data analysis	CaVEMan (Jones et al., 2016) and Pindel (Raine et al., 2015) algorithms were used for mutation calling, published methods. Filtering algorithms used were previously described in Ellis et al., (2021) and the beta-binomial and exact-binomial filters in Coorens et al. (2021). Structural variants were called using a published algorithm, GRIDDS v2.9.4 (Cameron et al., 2017). Copy number changes were called using Allele-Specific Copy number Analysis of Tumours (ASCAT) algorithm (Van Loo et al., 2010) as part of the ascatNGS package (Raine et al., 2016) (https://github.com/ Crick-CancerGenomics/ascat). A bespoke filtering algorithm - ascatPCA - was used to reduce the number of false-positive calls that can arise when analysing genome sequences from normal tissue (https://github.com/hj6-sanger/ ascatPCA). Custom mutation clustering algorithm using N-dimensional Dirichlet Process (NDP) was used, as well as custom tree building algorithm using the pigeonhole principle (both previously described in the methods in Brunner et al. (2019). R package dndscv v0.1.0 was used for selection analyses.

For manuscripts utilizing custom algorithms or software that are central to the research but not yet described in published literature, software must be made available to editors and reviewers. We strongly encourage code deposition in a community repository (e.g. GitHub). See the Nature Portfolio guidelines for submitting code & software for further information.

Data

Policy information about availability of data

All manuscripts must include a data availability statement. This statement should provide the following information, where applicable:

- Accession codes, unique identifiers, or web links for publicly available datasets
- A description of any restrictions on data availability
- For clinical datasets or third party data, please ensure that the statement adheres to our policy

Data availability
Whole genomes and exome sequencing data have been deposited in the European Genome–phenome Archive (EGA) (https://ega-archive.org/). WGS data have been deposited with EGA accession number EGAD00001015430 and exome sequencing data have been deposited with accession number EGAD00001015431. Existing DNA sequencing datasets from the liver of subjects with steatotic liver disease used in the study are deposited in EGA with accession code: EGAD00001006255. The crystal structure of native human A1AT was obtained from the RCSB Protein Data Bank (https://www.rcsb.org/structure/1qlp).

Code availability
Previously generated code that was used in this work was obtained from GitHub: Single-nucleotide substitutions were called using the CaVEMan algorithm, v.1.15.1 (https://github.com/cancerit/CaVEMan); small insertions and deletions were called using the Pindel algorithm, v.3.7.0 (https://github.com/cancerit/cgpPindel); selection analysis was performed using the dNdScv package, v.0.0.1.0 (https://github.com/im3sanger/dndscv). Newly generated code underpinning the downstream analyses is also available on GitHub (https://github.com/nataliabrz/positive_selection_in_A1AT_deficiency) and the specific versions (v1.0.0) are captured on Zenodo55. Whole genome and exome somatic mutation calls necessary to reproduce the analyses in the GitHub repository are available on Mendeley Data (https://data.mendeley.com/datasets/vhybvj2g9p/1)56.

Research involving human participants, their data, or biological material

Policy information about studies with human participants or human data. See also policy information about sex, gender (identity/presentation), and sexual orientation and race, ethnicity and racism.

Reporting on sex and gender	Patient sex was reported in the demographics information presented in Supp. Table 1. This was determined by self-reported patient information from the clinical record.
Reporting on race, ethnicity, or other socially relevant groupings	Patient ethnicity was reported in the demographics information presented in Supp. Table 1. This was determined by the research team based on previous clinical information and clinical data.
Population characteristics	Participants were included with chronic liver disease due to alpha-1 anti-trypsin deficiency or haemochromatosis on liver explant histology. Full demographic details are presented in Extended data Figure 1 and Supplementary Table 1.
Recruitment	Participants were identified through interrogation of the Cambridge University Hospitals NHS Foundation trust liver transplant database. Patients with specific liver disease aetiology requiring liver transplantation and with available liver tissue were included in the study. There is no self-selection bias
Ethics oversight	All liver samples were collected with written informed consent from Addenbrooke's Hospital, Cambridge, UK, according to procedures approved by Local Research Ethics Committees of Northern Ireland (20/NI/0109, 16/NI/0196).

Note that full information on the approval of the study protocol must also be provided in the manuscript.

Field-specific reporting

Please select the one below that is the best fit for your research. If you are not sure, read the appropriate sections before making your selection.

☒ Life sciences ☐ Behavioural & social sciences ☐ Ecological, evolutionary & environmental sciences

For a reference copy of the document with all sections, see nature.com/documents/nr-reporting-summary-flat.pdf

Life sciences study design

All studies must disclose on these points even when the disclosure is negative.

Sample size	No sample size calculation was performed before analyses. 5 patients with A1AD and 5 patients with haemochromatosis were included due to availability of liver tissue taken at liver transplantation.
Data exclusions	No data were excluded.
Replication	We analysed 5 people with each disease sub-group for the genomic analyses. All in vitro experiments were replicated at least three times, as detailed in each figure legend.
Randomization	No randomisation was performed as patients were recruited by the presence of explant histology evidence of specific liver disease. Therefore randomisation is not relevant to this study.

Blinding	No blinding was performed, other than blinded reporting of the liver sample histopathology, where clinical and genomic data was not revealed to the specialist liver histopathologist.

Reporting for specific materials, systems and methods

We require information from authors about some types of materials, experimental systems and methods used in many studies. Here, indicate whether each material, system or method listed is relevant to your study. If you are not sure if a list item applies to your research, read the appropriate section before selecting a response.

Materials & experimental systems

n/a	Involved in the study
☐	☒ Antibodies
☐	☒ Eukaryotic cell lines
☒	☐ Palaeontology and archaeology
☒	☐ Animals and other organisms
☒	☐ Clinical data
☒	☐ Dual use research of concern
☒	☐ Plants

Methods

n/a	Involved in the study
☒	☐ ChIP-seq
☒	☐ Flow cytometry
☒	☐ MRI-based neuroimaging

Antibodies

Antibodies used	ab9373, Abcam. Full length α1-antitrypsin human. Lot: 1051380-2. 1:200 ELISA<br>a9044, Sigma-Aldrich. HRP mouse secondary. 1:20,000 ELISA<br>MA5-15521, Thermo Fisher Scientific. Human α1-antitrypsin (peptide fragment 40-184 amino acids). Lot.: ZC4270403. 1:1000 SDS WB<br>A0409, Sigma-Aldrich. Full length human α1-antitrypsin. Lot: Unknown. 1:1000 Native-PAGE WB<br>HM2289, Hycult BIotech. human α1-antitrypsin polymer specific mAb2C1. Lot: 27047M0619-A. 1:500 Native-PAGE WB<br>mAb3C11, Lomas Laboratory. human α1-antitrypsin. Lot: Unknown. 1:200 undiluted hbridoma culture medium - ELISA<br>LiCor IRDyes: Goat anti mouse 680LT / 680LT goat anti rabbit / 800CW goat anti mouse / 800CW goat anti rabbit. 1:2000 of 0.1mg/ml stock - WB
Validation	ab9373 –Validated by ELISA using purified alpha-1-antitrypsin protein as a substrate (isolated from patient plasma to produce a single protein band when separated on SDS-PAGE).<br>MA5-15521 –Validated in extended figure 6A, reactive to a protein species corresponding to the size of the A1AT coding sequence transfected. No protein band detected in lysates of untransfected cells.<br>A0409 –Native PAGE western blot showed no reactivitytowards lysate of untransfected cells (data available upon request) and produced a banding pattern of M-and Z-A1AT (Fig4D, upper-left) corresponding to that observed from human plasma and intra-hepatic inclusions respectively (PMID: 33087346).<br>HM2289 –Validated by Lomas lab in PMID: 20583215.<br>mAb3C11–Commercialised as HM2358. Validated by Lomas lab in PMID: 25462157.

Eukaryotic cell lines

Policy information about cell lines and Sex and Gender in Research

Cell line source(s)	CHO-k1 (chinese hamster ovary. Female) cell line was purchased from Clontech and COS7 cells were purchased from MERCK. Stable cell lines were generated by following the manufacturer's instructions (PMID: 23197448).
Authentication	None.
Mycoplasma contamination	Cell lines were routinely checked for mycoplasma contamination and always found to be negative.
Commonly misidentified lines<br>(See ICLAC register)	No commonly misidentified cell lines were used in this manuscript.

Plants

Seed stocks	No plants were harmed in the making of this paper
Novel plant genotypes	No novel plants were generated
Authentication	No plants required authentication
