# Supplementary figures and images for: Selection for somatic escape variants in SERPINA1 in the liver of patients with alpha-1 antitrypsin deficiency
Source: Nat Genet. 2025 Mar 10;57(4):875–83. doi: 10.1038/s41588-025-02125-1 (PMC11985350; doi:10.1038/s41588-025-02125-1)

# Brzozowska - Uncropped immunoblots

**Fig. 4d**

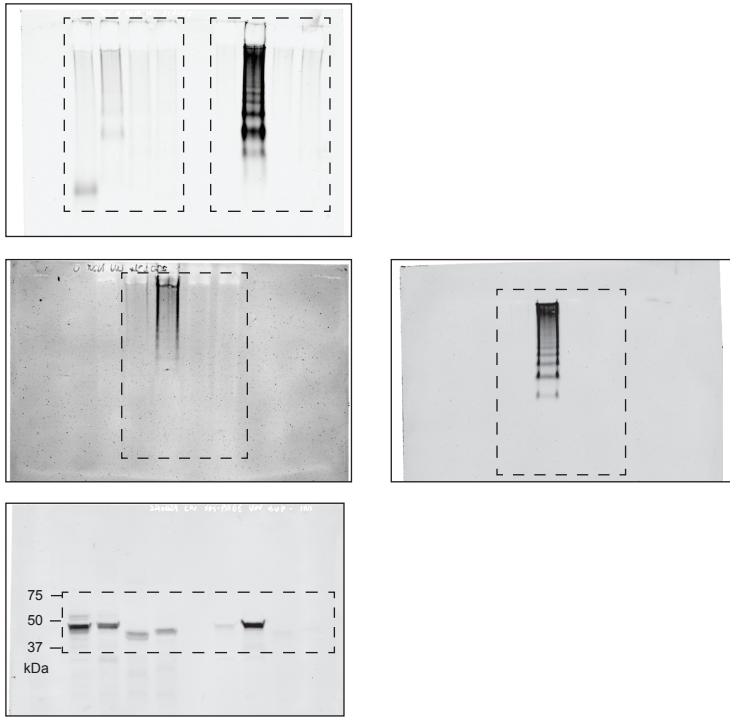

**Fig. 4g**

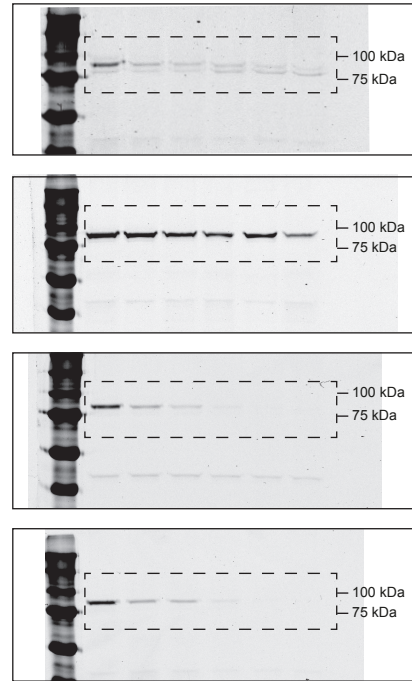

**Fig. 4h**

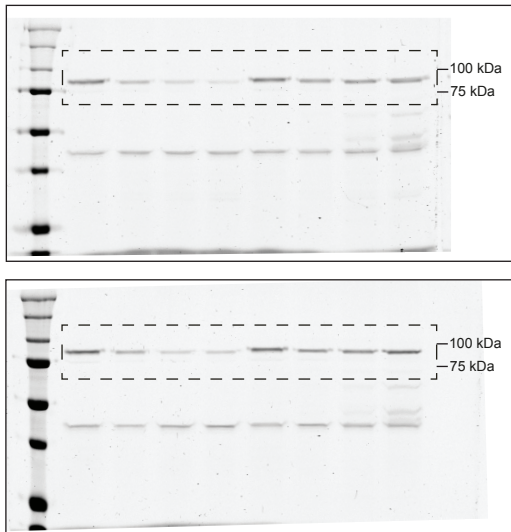

**Ext. Fig. 6A**

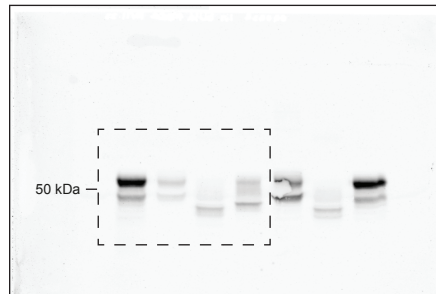

**Ext. Fig. 6C**

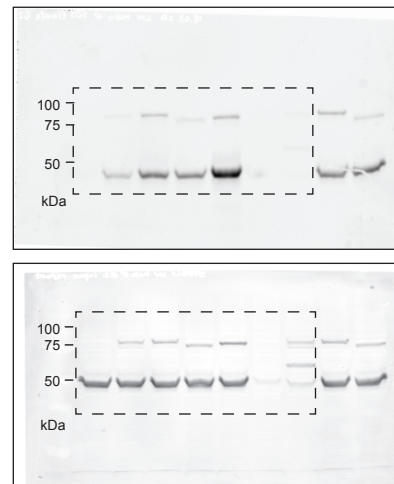

Supplement: Supplementary file 3 — Supplementary figure: full scans of immunoblots used in the indicated figures, including molecular-weight markers. [file 41588_2025_2125_MOESM3_ESM.pdf]
